# The Effect of Organic Trace Mineral Supplementation in the Form of Proteinates on Performance and Sustainability Parameters in Laying Hens: A Meta-Analysis

**DOI:** 10.3390/ani13193132

**Published:** 2023-10-07

**Authors:** Laurann Byrne, Stephen Ross, Jules Taylor-Pickard, Richard Murphy

**Affiliations:** 1 Alltech Biotechnology Centre, Summerhill Road, A86 X006 Dunboyne, Irelandrmurphy@alltech.com (R.M.); 2 Alltech E-CO2, Ryhall Road, Stamford PE9 1TZ, UK

**Keywords:** organic trace minerals, proteinate, meta-analysis, laying hen, carbon footprint, sustainability

## Abstract

**Simple Summary:**

This work aims to highlight the importance of mineral form on production performance and egg quality traits using meta-analysis. Sustainability impacts, which focused on the carbon footprint of egg production, were also assessed. Results demonstrated that the inclusion of organic trace minerals, in the form of proteinates, positively impacted key parameters such as hen-day production, feed conversion ratio, egg mass, egg weight, and egg loss. Eggshell parameters such as thickness, strength, weight, and eggshell percentage were also higher, and Haugh unit was 1 point greater with the inclusion of proteinate minerals.

**Abstract:**

The effect of supplementing organic trace minerals (OTM), in the form of mineral proteinates (Bioplex^®^ Cu, Fe, Mn, and Zn, Alltech Inc., Nicholasville, KY, USA), in the diets of laying hens was examined using Comprehensive Meta-Analysis (CMA) statistical software. The impact on production performance, egg quality traits, and sustainability parameters related to the carbon footprint of egg production was assessed. Data were obtained from 32 global studies, comprising 107 dietary assessments of 30,992 laying hens. Overall pooled effect size (raw mean difference) of production performance when dietary organic trace minerals were supplemented either in basal diets, partial replacement of inorganic trace minerals (ITM), or total replacement of ITM, indicated that use of Bioplex minerals resulted in 2.07% higher hen-day production (HDP), whilst feed conversion ratio (FCR) was lower by 51.28 g feed/kg egg and 22.82 g feed/dozen eggs, respectively. For egg quality traits, daily egg mass was 0.50 g/hen/day higher and egg weight was 0.48 g per egg greater when Bioplex minerals were incorporated in the diet. The mean difference in egg loss was −0.62%. Eggshell thickness was greater by 0.01 mm, and a higher eggshell strength of 0.14 kgf was observed. Eggshell weight was heavier by 0.20 g, eggshell percentage was higher by 0.15%, and Haugh unit was 1 point higher (0.89). We also carried out a meta-regression of the effects of the study factors (location, year of study, hen breed/strain, age of hens, number of hens, and study duration) on the overall pooled effect size of the production performance and egg quality traits in response to supplementary OTM inclusion, and it indicated that certain factors had a significant (*p* < 0.05) impact on the results. Finally, a life cycle assessment (LCA) model was selected to evaluate the impact of feeding organic trace mineral proteinates on the carbon footprint (feed and total emission intensities) of the egg production using the data generated from the meta-analysis. Results showed that the inclusion of OTM proteinates resulted in an average drop in feed and total emission intensities per kg eggs of 2.40% and 2.50%, respectively, for a low-global-warming-potential (GWP) diet and a drop of 2.40% and 2.48% for feed and total emissions, respectively, based on high-GWP diet. Based on the overall results, the inclusion of organic trace mineral proteinates in layer diets can benefit production performance and egg quality traits while contributing to a lower carbon footprint.

## 1. Introduction

Mineral nutrition plays a key role in poultry production; however, many diets are routinely formulated based on outdated data [1,2,3]. Cognisant of optimising the genetic potential of modern breeds, research on mineral supplementation has become a necessity [4]. Inorganic mineral salts are regularly overformulated to ensure adequate levels are included to meet dietary requirements; however, uptake and utilisation are limited, resulting in poor performance results [5,6,7]. Mineral levels have also come under additional scrutiny from an environmental perspective, and providing more than required will become a less acceptable practice [8,9]. Organic trace minerals (OTM) are noted to be more bioavailable than inorganic sources, with enhanced performance and lower mineral excretion being reported [10]. These responses can have positive environmental benefits, but different forms of OTM exist, varying greatly in efficacy [10,11,12,13]. As poultry genotypes have evolved, birds are required to produce more with less inputs; however, the underlying physiological mechanisms for mineral uptake remain the same. High-performance breeds may exceed standard mineral absorption mechanisms, and the only way to achieve a constant mineral supply at a cellular level is by increasing dietary levels or using sources with higher relative bioavailability [13]. Nutritionists have started to redefine the mineral levels used in poultry diets for several reasons, including decreasing feed costs while improving performance, enhancing the sustainability of poultry production, and lowering antagonist interactions with other dietary nutrients. The formulation of such diets, that target precision nutrition, will require the most bioavailable sources of minerals.

Numerous trials under various conditions have been conducted globally to investigate the total replacement of Cu, Fe, Mn, and Zn in their inorganic salt form with lower amounts of the same minerals in proteinate form. Results have indicated that the use of lower OTM proteinate amounts could not only maintain or optimise growth and reproductive performance, but also lower the mineral content of excreta [1]. With an abundance of published trial data in existence, confusion or misinterpretation can occur. Employing methods such as meta-analyses to systematically combine data from multiple trials provides evidence-based conclusions to aid in alleviating such issues. The objective of this study was to apply this type of statistical procedure to examine the impact of supplementing OTM in the form of proteinate minerals on the production performance and egg quality attributes of laying hens. Carbon footprint (CFP; total GHG emissions associated with the production of a functional unit of output) was also calculated using the meta-analysis results, enabling the effect of feeding proteinate trace minerals on the CFP of egg production to be quantified. A scenario simulation was also developed using a life cycle assessment (LCA) model to provide a method for quantifying the CFP of livestock products from cradle to farmgate [14].

## 2. Materials and Methods

### 2.1. Literature Search and Selection Criteria

**Figure 1:**
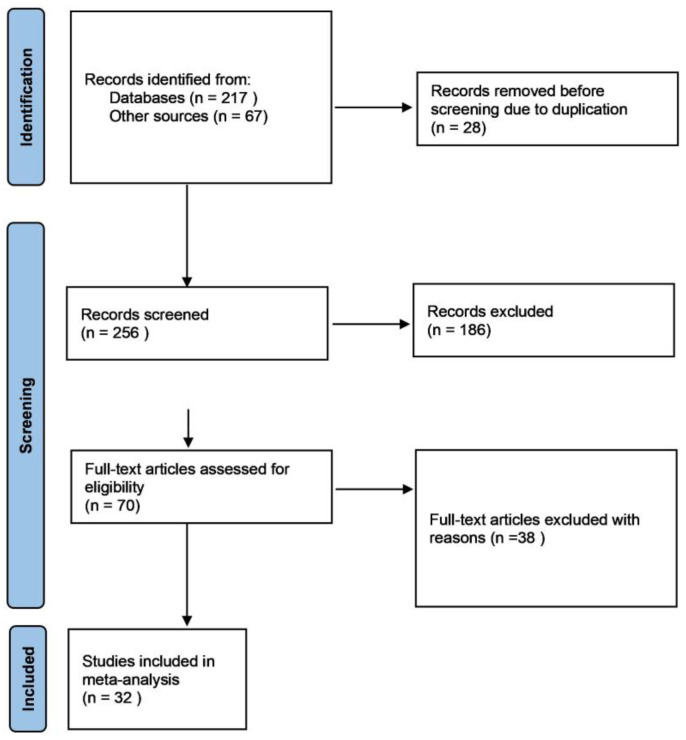
PRISMA flow diagram describing the literature search strategy and study selection for the meta-analysis.

A comprehensive data search was carried out using Google Scholar, Mendeley, PubMed, Web of Science, CAB Direct, and Scopus databases to obtain peer-reviewed articles evaluating the effect of a commercial organic trace mineral product (Bioplex Cu, Fe, Mn, and Zn, Alltech Inc., Nicholasville, KY, USA) on layer performance. Furthermore, the company’s internal bibliographic database was searched to retrieve published and unpublished trial reports presented in a Ph.D. dissertation or at international scientific conferences. Keywords used for the digital search included “layers”, “poultry”, “organic trace element”, “organic trace mineral”, “Bioplex”, “total replacement”, partial replacement”, and “layer performance”. The literature search and study selection applied in this meta-analysis, carried out according to the Preferred Reporting Items for Systematic Reviews and Meta-Analyses (PRISMA) Statement [15,16,17], are reported in Figure 1. Two reviewers, working independently, screened each record and collected the data. The final literature search to obtain relevant data was conducted in February 2023 and there was no date restriction imposed on the literature search, to cover the entire duration that the OTM products have been investigated in layers.

A total of 70 research articles were initially identified for screening and were subjected to the following criteria: (1) the study was reported in English; (2) the trial was conducted in layers and adequate randomisation of birds into treatments was reported; (3) the study contained at least one negative control (basal diet) and/or positive control (inorganic trace mineral diet) and a diet supplemented with Bioplex^®^ as the organic trace mineral product; (4) the trace mineral dosage application rate and feeding duration were reported; (5) information describing the study factors of the experiments was provided or available upon request from authors; and (6) information on one or more performance parameters and egg quality attributes was reported or available upon request from authors. Post-initial screening, 32 trials were selected for inclusion in the meta-analysis; they are outlined in Table 1.

### 2.2. Data Extraction

Using the data extracted from the 32 selected trials, a database was developed consisting of 107 comparisons of basal or inorganic trace mineral supplemented diets vs. organic trace mineral supplemented diets. Basal diets represented a comparison of basal diets vs. basal + supplementary OTM. Partial replacement (PR) represented a comparison of basal diets + supplementary inorganic trace minerals (ITM) vs. basal diets + partial replacement of supplementary ITM with OTM. Total replacement (TR) represented a comparison of basal diets + supplementary ITM vs. basal diets + total replacement of supplementary ITM with OTM.

Trace mineral concentration, form (ITM/OTM), and type (Cu, Fe, Mn, and Zn) were recorded (Table 2). Mean OTM levels from all studies assessed were lower than mean ITM inclusion rates for all minerals, indicating that lower OTM levels can be included without a detrimental impact on performance parameters. Data on production performance parameters (feed intake (g/day/hen), hen-day production (%), FCR (g feed/kg egg), and FCR (g feed/dozen eggs)) and egg quality traits (egg mass (g/hen/day), egg weight (g), egg loss (%), eggshell thickness (mm), eggshell strength (kgf), eggshell weight (g), eggshell percentage (%), and Haugh unit) were also extracted (Table 3). The measure of variance was documented as standard deviation (SD). Additionally, data on various study factors including location, year of study, hen breed/strain, age of hens, number of hens and study duration were obtained.

### 2.3. Statistical Analysis

Comprehensive Meta-analysis software (version 3, Biostat Inc., Englewood, NJ, USA) was utilised to analyse the effect of treatment comparisons in a random-effects model. A random-effects model allowed the true effect to vary from study to study and include between-study variability (true heterogeneity) as well as sampling error [47]. Raw mean difference (RMD) and standardised mean difference (SMD) at a 95% level of confidence interval (CI) were used to estimate the effect size of dietary OTM supplementation on layer performance parameters and egg quality variables. Effect size estimates (RMD and SMD) of all trial comparisons (OTM diets vs. basal and ITM diets) on performance variables were declared significant when *p* ≤ 0.05, and a tendency for effect was observed when 0.05 < *p* ≤ 0.10. Furthermore, sub-databases were created to perform a meta-analysis of subgroups, evaluating how study factors influence the response of selected production performance and egg quality variables (feed intake, HDP, FCR, egg weight, egg loss, and eggshell strength) to OTM supplementation. Study factors consisted of location (Africa, Europe, North America, Oceania, South America, Asia), year of study, hen breed/strain (Black Harco, Bovans, Brown Yaffa, H&N Brown Nick, Hy-Line, ISA Brown, Jinghong, Roso SL hybrid, White Leghorn, Lohmann), age of hens, number of hens, and study duration.

### 2.4. Heterogeneity

Variation across studies was assessed using the *I*^2^ statistic and the associated significance level of chi-squared statistic [47]. *I*^2^ values of <25%, 25 to 50%, and >50% suggest low, moderate, and high heterogeneity, respectively [48].

### 2.5. Publication Bias

Funnel plots were created for each outcome to assess the risk of bias in the studies included in the meta-analysis. The standard error of the observed outcomes as a predictor was used to check for funnel plot asymmetry with symmetrical distribution of studies around the calculated mean difference (MD), indicative of no bias risk. The potential risk of bias was identified through an observed asymmetrical distribution around MD and confirmed by Egger’s test with *p* < 0.05 indicative of the presence of bias in the funnel plot [49].

### 2.6. Life Cycle Assessment

#### 2.6.1. Scope

**Figure 2:**
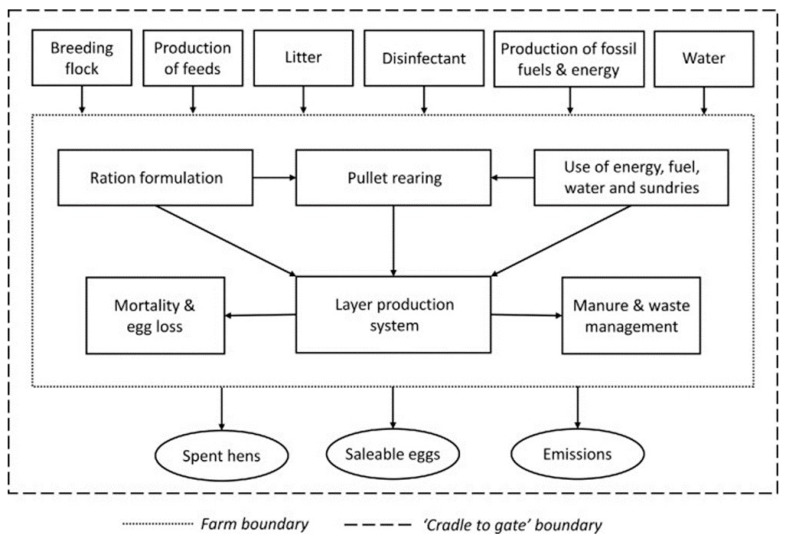
The structure and system boundary of the egg production system considered in the life cycle assessment (adapted from Salami et al., 2022 [53]).

Life cycle assessment (LCA) simulation modelling was conducted to assess the impact of feeding organic trace minerals on the carbon footprint (CFP) of layer production. The system boundary encompassed all processes involved in layer production up to finished birds being ready to leave the farm (i.e., cradle to farmgate). As such, modelling accounted for both pre-farm supply chain emissions, including the production burden of purchased feeds and raw materials, and the on-farm emissions from layer production and litter management. Subsequent emissions attributed to post-farm processing, packaging, or transport of products beyond the farm gate were not considered, consistent with previous LCA studies analysing egg production systems [50,51,52,53]. A flow diagram describing the LCA system boundary is outlined in Figure 2.

Outputs from the system are spent hens, saleable eggs, and litter. All litter was exported to be land-spread as fertiliser and allocated a carbon credit in the model through system expansion; thus, the eggs and spent hens were considered co-products of the system. The functional unit of the layer assessment was the primary egg product from the system. Emission intensity was presented using three functional units, kg CO_2_-eq/dozen eggs, g CO_2_-eq/egg, and kg CO_2_-eq/kg eggs, leaving the farm gate. The partitioning of total emissions between eggs and sold spent hens was determined based on economic allocation. The assessment period was defined as the birds’ lay period (62 weeks), and all scenarios started with a flock of 100,000 birds on the farm.

#### 2.6.2. Production System and Scenarios

Production systems modelled in this LCA were based upon an average conventional European housed layer system. Four scenarios were defined within this production system: a baseline scenario (i.e., without OTM supplementation) and an intervention scenario (i.e., with OTM supplementation) for two different diets defined by the level of global warming potential (GWP) attributed to each. The low-GWP diet consisted of low-level soybean meal (SBM) inclusion, whereas the high-GWP diet consisted of a higher inclusion level of SBM (Supplementary Table S1). For the low-GWP diet, soybean meal inclusion rates were 15.21%, 15.34%, 6.52%, and 3.60% for the pre-lay, early-lay, mid-lay, and late-lay stages, respectively. For the high-GWP diet, soybean meal inclusion rates were 15.21%, 24.35%, 9.35%, and 3.60% for the pre-lay, early-lay, mid-lay, and late-lay stages, respectively. Feed ingredients were consistent in the baseline and OTM diets, other than the OTM supplementation. All feed ingredients were assumed purchased and delivered onto the layer farm. Birds in all scenarios were managed on 4 successive formulated rations (pre-, early-, peak- and late-lay diets) according to industry practice (Supplementary Tables S1 and S2). Pullets were delivered to the farm after a 16-week rearing period and placed in a flock of 100,000 birds. The subsequent laying period lasted 62 weeks equally across all systems after a 2-week pre-lay period.

#### 2.6.3. Inventory Analysis

Input data on production system parameters were averaged from 300 commercial European layer farm environmental assessments conducted by Alltech E-CO_2_ of the same system type over a two-year period (2018–2020) (Alltech E-CO_2_, Stamford, UK). Baseline metrics on entry into the lay cycle were measured as: laying cycle (62 weeks), hen-day production (85%), eggs produced per bird (369), egg weight (65 g), total egg mass per hen (23.99 kg), mortality (3.5%), egg loss (1.5%), total feed per hen (50.35 kg), and FCR (2100 g/kg eggs). Bird age milestones were: on entry (17 weeks), point of lay (19 weeks), end of lay/life (81 weeks), and total laying period (62 weeks). In total, there were 4 phase diets and the period of each included the pre-lay (2 weeks), early-lay (26 weeks), peak-lay (19 weeks), and late-lay (16 weeks) periods. Production performance parameters and egg quality traits when managed in the baseline and OTM scenarios are presented in Supplementary Table S3. The OTM results were derived using the observed results from the meta-analysis, applying the higher relative percentage values observed in production performance parameters compared to the baseline. Commercial data were also used to obtain average input values for general resource use (e.g., bedding, fossil fuels, electricity, water use, and disinfectant). The study assumed that all litter was exported for use as organic fertiliser after being stored on-farm until the flock clear-out. All viable birds were sold at the production period end, and dead birds were assumed sent to a rendering plant.

#### 2.6.4. Impact Assessment

The Alltech E-CO_2_ Poultry EA™ (layer) model (Alltech E-CO_2_, Stamford, UK), an industry-specific layer CFP calculator, was employed to conduct the environmental impact assessment. Independently accredited to the LCA international standards PAS:2050 (BSI, 2011) and ISO 14067 (ISO, 2018), this bespoke model has been utilised in commercial layer CFP assessments since 2014. The model follows the Intergovernmental Panel on Climate Change (IPCC) guidelines for tier 2 methodology for on-farm emissions and has the ability to implement case-specific farm data at a tier 3 level [54]. Nitrogen excreted by hens was estimated from the total feed intake per bird, the weighted protein content of the formulated rations, and the estimated percentage of dietary nitrogen excreted by the animal, following IPCC (2006) [54]. Emissions arising from manure and litter management (i.e., CH_4_, direct and indirect N_2_O) were also estimated using tier 2 equations from IPCC (2006) [54].

Publicly available data from the software FeedPrint (v2020.00), developed by Wageningen University and Research (WUR), The Netherlands, were used to estimate emissions embedded in the production, processing, and supply of purchased feeds [55]. Data sourced from the WUR model were noted to be derived according to PAS:2050, with an economic allocation of co-products; therefore, they were considered directly compatible with the methodology employed in the present study. Feed ingredients were assumed to be typical of the European market mix, and SBM was assumed to originate from South America (including emissions associated with land-use change). Emission factors derived for each formulated ration are outlined in Supplementary Table S2.

Embedded emissions in pullets placed on the farm were taken to be 4.06 kg CO_2_-eq per bird, as retrieved from Alltech’s E-CO_2_ commercial database. Emission factors for all electricity, fossil fuels, and transportation (i.e., of chicks, feed, and dead birds’ removal) were sourced from the UK Department for Environment, Food and Rural Affairs, DEFRA (2020) [56]. Exported litter was assumed destined to be used as fertiliser, and accordingly, a carbon credit was applied via the method of system expansion. Coefficients for nutrient content and availabilities in layer litter were described by DEFRA (2010) [57], and data on the avoided emissions of inorganic fertiliser production were provided by Hoxha and Christensen [58].

Layer production life cycle emissions were calculated using GHG species’ particular global warming potentials for 100 years (GWP_100_), stated as 25 and 298 times greater than CO_2_ for CH_4_ and N_2_O, respectively [59]. Feed emission intensity (i.e., kgCO_2_-eq attributed to the feed use per functional unit) and total emission intensity (i.e., total kgCO_2_-eq per functional unit) of the production system were subsequently estimated for each of the four scenarios for the production life cycle of 100,000 birds placed in the layer system.

## 3. Results and Discussion

### 3.1. Study Characteristics

The 32 studies selected for this meta-analysis are outlined in Table 1. Studies were obtained from 14 countries: USA (5), Canada (3), Brazil (6), Peru (2), Nigeria (2), Turkey (2), China (2), Iran (1), Australia (2), Romania (1), Switzerland (1), Slovakia (1), Poland (1), and Czech Republic (3) over a 21-year time period (1999–2020). A total of 30,992 laying hens were involved in the 107 dietary comparisons comprised of Basal/ITM vs. Bioplex supplementation.

Table 2 outlines the trace mineral concentrations supplemented in layer diets and details the treatment comparisons involving total replacement of ITM with OTM (Bioplex Cu, Fe, Mn, and Zn). Results clearly show that supplementation levels of OTM are lower for all minerals compared to ITM levels. Many studies have confirmed that lower inclusion levels of OTM proteinates are possible due to their enhanced bioavailability and absorption, resulting in a lower requirement without negatively impacting animal health or performance [1,9,28,32,36,37,60,61,62,63,64,65,66,67,68,69].

Considerable variation exists in the dataset of production performance and egg quality parameters included in the meta-analysis (Table 3). The global diversity of studies can account for this variation in the dataset, and a wide representation enables meaningful conclusions to be drawn from this meta-analysis.

### 3.2. Production Performance and Egg Quality

The results of the overall pooled effect size of production performance and egg quality traits when dietary OTM were supplemented either in basal diets, partial replacement for ITM, or total replacement for ITM are presented in Table 4. Corresponding forest plots for each variable are presented in Supplementary Figures S1–S12. For feed intake, no effect was observed with OTM supplementation (*p* > 0.05) (Table 4, Supplementary Figure S1). HDP was significantly higher (RMD = +2.07), and FCR was significantly lower (RMD = −51.28 g feed/kg egg), in OTM-supplemented flocks (Table 4 and Supplementary Figures S2–S4). Compared to the control, the overall pooled effects of OTM on HDP was +2.48% higher. FCR (g feed/kg egg) was 2.39% lower.

For egg quality traits, incorporating OTM resulted in higher egg mass (RMD = +0.50 g/hen/day) and egg weight (RMD = +0.48 g) (Table 4 and Supplementary Figures S5 and S6). The mean difference in egg loss was −0.62%, which equates to a 39.24% lower egg loss (Table 3 and Supplementary Figure S7). Eggshell thickness was significantly higher (RMD = +0.01 mm, *p* < 0.001), by 2.94%, and eggshell strength was also significantly higher (RMD = +0.14 kgf, *p* < 0.001), by 3.84% (Table 4 and Supplementary Figures S8 and S9). Significantly higher values (*p* < 0.001) were also noted for both eggshell weight (RMD = +0.20 g), which was 2.39% higher, and eggshell percentage (RMD = +0.15%), which was 1.76% higher (Table 4 and Supplementary Figures S10 and S11). Haugh unit was also significantly higher (*p* < 0.001), by 1 point (RMD = +0.89) (Table 4 and Supplementary Figure S12).

**Figure 3:**
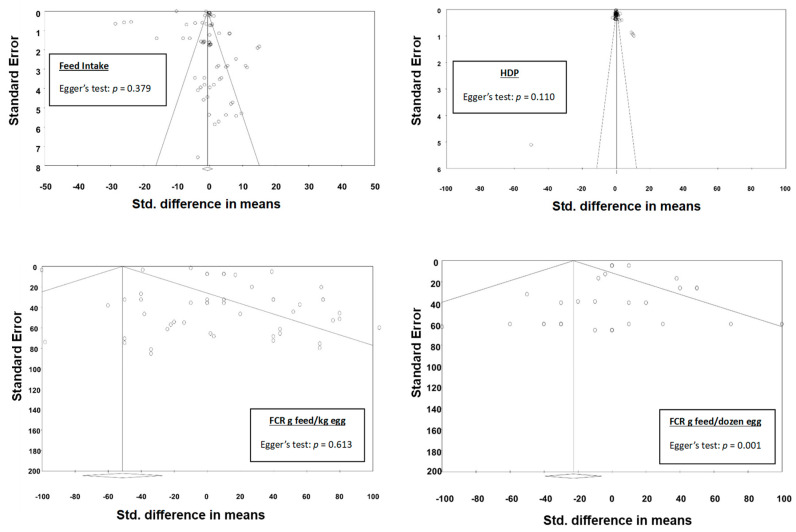
Funnel plots of standardised mean differences against their inverse standard errors and the associated significance (*p*-value for Egger’s test) for testing the publication bias of studies included in the meta-analysis for production performance parameters: feed intake, hen-day production, and feed conversion ratio. Open circles represent individual study comparisons included in the meta-analysis.

**Figure 4:**
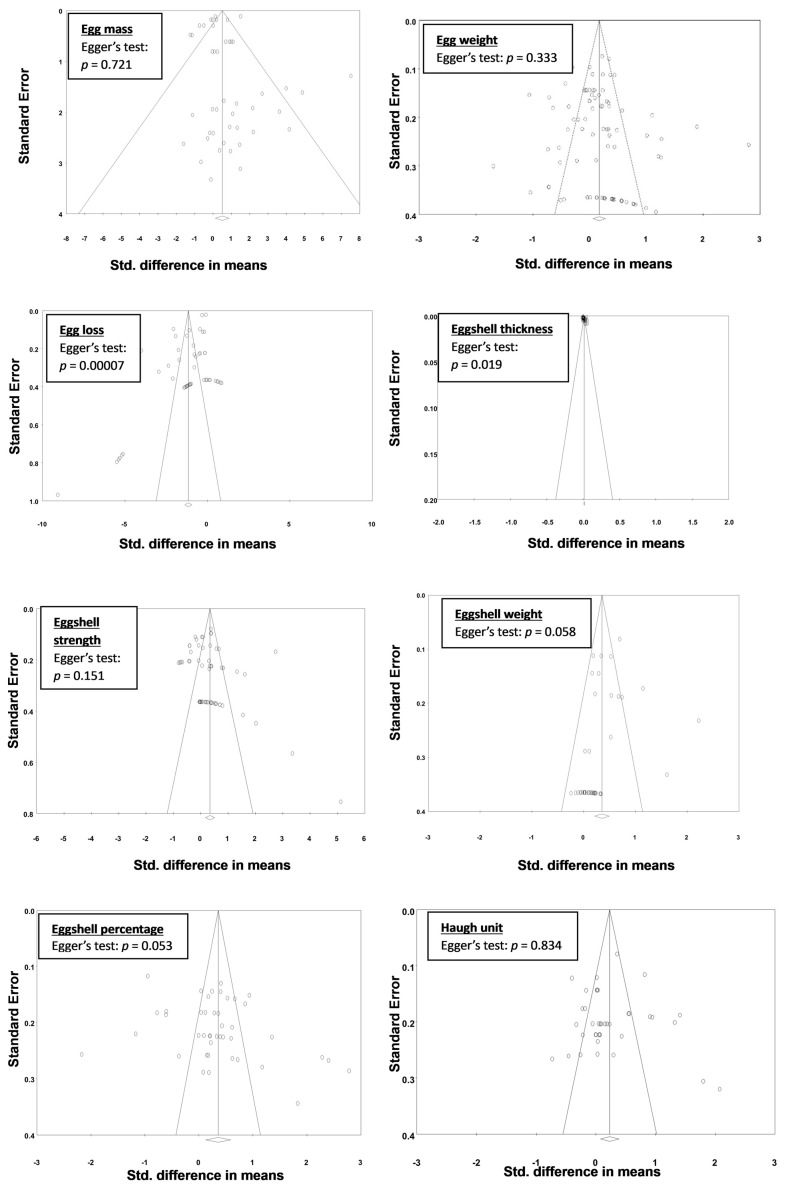
Funnel plots of standardised mean differences against their inverse standard errors and the associated significance (*p*-value for Egger’s test) for testing the publication bias of studies included in the meta-analysis for egg quality traits: egg mass, egg weight, egg loss, eggshell thickness, eggshell strength, eggshell weight, and eggshell percentage. Open circles represent individual study comparisons included in the meta-analysis.

Visual inspection of the funnel plots for all experiments selected for meta-analysis indicates the absence of bias due to the symmetrical distribution of the weighted mean difference in most experiments around the standard error (Figure 3 and Figure 4). Additional analyses using Egger’s test returned nonsignificant values for all parameters with the exception of egg loss, eggshell thickness, and FCR (g/dozen eggs), which returned *p* values of <0.05, indicating possible publication bias; however, the observed asymmetry could be explained by considering meta-regression residuals [70].

Table 5 outlines the effect size of production performance and egg quality traits when dietary OTM were supplemented in basal diets, whereas Table 6 consists of effect size data when OTM were supplemented as total replacement for ITM. Comparing the resultant data in each table for all parameters, no effect on feed intake was observed with OTM supplementation (*p* > 0.05) in either scenario. HDP was significantly higher when OTM supplementation was included as total replacement of ITM (RMD = +1.52) (Table 6), and tended towards significance (*p* = 0.017) when supplemented in basal diets (Table 5). FCR was significantly lower (*p* < 0.001) with total replacement (RMD = −73.09 g feed/kg egg) (Table 6). Examining the data obtained for egg quality traits, totally replacing ITM with OTM resulted in higher egg mass (RMD = +0.50 g/hen/day) and significantly higher (*p* < 0.001) egg weight (RMD = +0.76 g) (Table 6). The mean difference in egg loss was −0.57%, which equates to 41.30% lower egg loss (Table 6). Eggshell thickness was significantly greater (RMD = +0.01 mm, *p* < 0.001), by 3.13%, and eggshell strength was also significantly higher (RMD = +0.15 kgf, *p* < 0.001), by 3.99%. Significantly higher values (*p* < 0.001) were also noted for eggshell weight (RMD = +0.11 g) and Haugh unit (*p* < 0.05). Positive impacts were also seen when including OTM in basal diets, but a far greater number of highly significant (*p* < 0.001) results were found in studies when total replacement of ITM with OTM occurred. Significant heterogeneity (*p* < 0.001) was observed in all comparisons, except when the dataset was analysed for total replacement of ITM with OTM for eggshell weight (Table 5 and Table 6). Subgroup analysis was carried out to assess this further, with the meta-regression indicating effects from a number of study factors. This heterogeneity was expected, considering that the studies were performed in different countries under different production management systems.

Based on the results obtained for production performance and egg quality traits, incorporating OTM, in place of ITM, either partially or fully, has a positive impact on the metrics assessed. Previous publications have discussed the benefits of incorporating OTM in poultry nutrition, and the findings in this meta-analysis confirm such results [28,37,65,67].

### 3.3. Subgroup Analysis

Results from subgroup analysis of the effect of various study factors on the overall pooled effect size of production performance parameters and egg quality traits in response to supplementary OTM are presented in Table 7 and Table 8, respectively. The list of study factors is not exhaustive; other factors such as diet composition, housing, hygiene, genetics, and the type of production system may also contribute.

Feed intake, certain location, breed, and the age of hens had an impact (Africa (*p* < 0.001), Black Harco (*p* < 0.001), Brown Yaffa (*p* < 0.05), age (*p* < 0.05)) (Table 7). In relation to HDP, many of the study factors had a highly significant (*p* < 0.001) impact, including location, year of study, hen breeds, age of hens, number of hens, and study duration (Table 7). Key impacts on FCR (g feed/kg egg) included particular locations (Africa (*p* < 0.001), North America (*p* < 0.05), certain hen breeds (Bovans (*p* < 0.05), Brown Yaffa (*p* < 0.001), H&N Brown Nick (*p* < 0.05), Hy-Line (*p* < 0.05)), age of hens (*p* < 0.001), and study duration (*p* < 0.001)).

Three egg quality traits (egg weight, egg loss, and eggshell strength) were also assessed using a meta-regression of the effects of study factors on the overall pooled effect size in response to OTM supplementation (Table 8). For egg weight, only the hen breed (Bovans, Brown Yaffa, and H&N Brown Nick) had a significant effect (*p* < 0.05). For egg loss, the number of hens and the study duration, in addition to the hen breed (H&N Brown Nick, Hy-Line and Isa Brown), had a significant effect (*p* < 0.05). Location (North America) (*p* < 0.001), hen breed (Hy-Line, Isa Brown, and Jinghong) (*p* < 0.05), age of hens (*p* < 0.05), and study duration (*p* < 0.05) had a significant effect on eggshell strength.

Inclusion of a meta-regression ensures that additional factors of potential variance are assessed and incorporated into the overall meta-analysis findings, further reducing bias and explaining additional contributing factors of heterogeneity. From the meta-regression results in this study, it is clear that the factors assessed had a notable impact on overall results, demonstrating the importance of including extended methods of statistical analysis.

### 3.4. Simulated Environmental Impact

Metrics selected for quantifying the effect of supplementing OTM on the carbon footprint of egg production were feed emission intensity and total emission intensity, expressed as three functional units (emissions per dozen eggs, emissions per egg, and emissions per kg eggs). Feed emission intensities per kg eggs were lower in the OTM-supplemented diets for both the low-GWP and high-GWP diet scenarios, by an average of 2.40% (−0.04 and −0.05 kg CO_2_-eq/kg eggs, respectively) (Table 9). Emissions per egg were lower by 1.65% on average for both diets supplemented with OTM (−1.76 and −2.10 g CO_2_-eq/egg), and emissions per dozen eggs, on average, were 1.65% lower for both diets supplemented with OTM (−0.02 and −0.02 kg CO_2_-eq/ dozen eggs). Similarly, total emission intensity per kg eggs was lower by an average of 2.50% in the low-GWP diet and by 2.48% in the high-GWP diet (−0.05 and −0.07 kg CO_2_-eq/kg eggs, respectively). Emissions per egg were lower, on average, by 1.75% (low-GWP) and 1.73% (high-GWP) for both diets supplemented with OTM (−2.5 and −3.0 g CO_2_-eq/egg, respectively), and emissions per dozen eggs were lower by an average of 1.75% (low-GWP) and 1.73% (high-GWP) for diets supplemented with OTM (−0.03 and −0.04 kg CO_2_-eq/ dozen eggs). Previous LCAs for swine and poultry have also noted the appropriateness of the method for assessing the effect of specific feeding practices on the environmental performance of a farm’s product supplied to the market [71,72].

Trace minerals are required to support development and improve the productive performance of laying hens; however, the form that in which the trace mineral is supplemented in the diet plays a crucial role [10,11,28,36,37,67]. Mineral proteinates show higher retention rates and relative bioavailability values than inorganic trace minerals, and the results from this meta-analysis indicate that positive results on production performance and egg quality traits by incorporating OTM in layer diets. Sustainability impacts were also assessed, which focused on the carbon footprint of egg production and, the results indicate that lower feed emissions can be achieved by incorporating OTM.

## 4. Conclusions

With minerals including Cu, Fe, Mn, and Zn being essential for the growth and development of chickens and their involvement in various physiological processes, selecting the most bioavailable form is essential to optimise health and performance. The results from the meta-analysis demonstrate higher values for a number of key production performance parameters by using organic trace minerals, even at substantially lower inclusion rates, in the form of proteinates (Bioplex Cu, Fe, Mn, and Zn, Alltech Inc., Nicholasville, KY, USA). Hen-day production was greater by +2.07% and FCR was lowered by 51.28 g feed/kg egg and 22.82 g feed/dozen eggs, respectively. Positive impacts were also observed on egg quality traits, with egg mass greater by 0.50 g/hen/day and egg weight higher by 0.48 g per egg, on average. The mean difference in egg loss was lower by −0.62%, which equates to 39.24% less egg loss. Eggshell thickness was greater by 2.94% (0.01 mm), and a greater eggshell strength, by 3.84% (0.14 kgf), was observed. Eggshell weight was greater by 2.39% (0.20 g) and eggshell percentage was greater by 1.76% (0.15). Haugh unit was 1 point higher. With correct formulation, more cost-effective, environmentally sustainable feeds can be produced, resulting in a greater return on investment (ROI) and a lower carbon footprint.

## Figures and Tables

**Table 1:** Overview of studies included in the meta-analysis to evaluate the effects of supplementing organic trace minerals (Bioplex Cu, Fe, Mn, and Zn) in the diets of laying hens.

Reference	Study Location	Breed/Strain	Age of Hens (wk)	Number of Hens per Treatment	Treatment Comparison ^1^	Supplementary OTM/ITM	Study Duration (wk)
Murakami and Franco [18]	Brazil	Lohmann	32	160	Basal	Zn, Mn	20
Sara, Odagiu [19]	Romania	Roso SL hybrid	49	78	Basal	Zn, Mn, Cu	23
Schäublin, Wiedmer [20]	Switzerland	-	21	1872	TR	Cu, Fe, Mn, Zn	60
Nunes, Rossi [21]	Brazil	Hisex Brown	30	64	TR	Zn, Mn, Cu, Fe	12
MacIsaac, Anderson [22]	Canada	White Leghorn	55	72	Basal	Zn, Mn, Cu, Fe	12
Güçlü, Kara [23]	Turkey	Bovans	30	18	Basal	Cu	4
Leeson, Sefton [24]	Canada	Lohmann	18	360	TR	Zn, Mn, Cu	52
Sefton and Leeson [25]	Canada	Lohmann	36	36	TR	Zn, Mn, Cu, Fe	6.6
Ao, Pierce [26]	USA	Hy-Line Brown	16	96	TR	Zn, Mn, Cu, Fe	44
Revell, Zarrinkalam [27]	Australia	Hisex Brown	34	12	Basal, TR	Fe	6
Stefanello, Santos [28]	Brazil	Hy-Line W36	47	40	Basal, TR	Zn, Mn, Cu	15
Nolan [29]	USA	Hy-Line W36 and Hy-Line Brown	29	48	Basal, TR	Zn	36
Ceylan and Scheideler [30]	USA	Hy-Line W36	20	36	Basal	Zn, Mn	40
Carlos and Solano [31] (trial 1)	Peru	Hy-Line	27	4000	PR	Zn, Mn, Cu, Fe	10
Carlos and Solano [31] (trial 2)	Peru	Hy-Line	54	3525	PR	Zn, Mn, Cu, Fe	14
Boruta, Swierczewska [32]	Poland	Hy-Line	30	48	PR	Zn, Mn, Cu, Fe	40
Siske, Zeman [33] (trial 1)	Czech Republic	Isa Brown	17	213	PR	Zn, Mn	50
Siske, Zeman [33] (trial 2)	Czech Republic	Isa Brown	17	160	PR	Zn, Mn	55
Siske, Zeman [33] (trial 3)	Czech Republic	Isa Brown	53	320	Basal	Zn, Mn, Cu	19
Xavier, Rutz [34]	Brazil	Isa Brown	77	64	Basal	Zn, Mn	24
Ao, Paul [35]	USA	Hy-Line W36	17	84	TR	Zn, Mn, Cu, Fe	53
Ao, Pescatore [36] ^2^	USA	Hy-Line Brown	-	120	TR	Zn, Mn, Cu, Fe	80
Qiu, Lu [37]	China	Hy-Line White	50	135	TR	Zn, Mn, Cu, Fe	8
Yıldız, Cufadar [38]	Turkey	H&N Brown Nick	49	15	TR	Mn	12
Idowu, Ajuwon [39] ^2^	Nigeria	Brown Yaffa	-	60	Basal, TR	Zn	10
Idowu, Laniyan [40]	Nigeria	Black Harco	30	30	Basal, TR	Cu	10
Venglovska, Gresakova [41]	Slovakia	Lohmann Brown	23	24	Basal, TR	Mn	8
Torki, Akbari [42]	Iran	Lohmann LSL-Lite	42	30	Basal	Zn	8
Li, Zhang [43]	China	Jinghong-1	53	96	TR	Mn	10
Stringhini, Santos [44]	Brazil	Bovans White	60	96	PR, TR	Zn, Mn, Cu, Fe	20
Kocher, Kumar [45]	Australia	Isa Brown	64	70	TR	Zn, Mn, Cu, Fe	30
Fernandes, Murakami [46]	Brazil	Hy-Line W36	67	64	Basal	Zn, Mn	16

OTM: organic trace minerals (Bioplex Zn, Mn, Cu, and Fe); ITM: inorganic trace minerals. ^1^ Treatment comparison: Basal represents comparison of basal diets (conventional diets) vs. basal diets + supplementary OTM; PR (partial replacement) represents comparison of basal diets + supplementary ITM vs. basal diets + partial replacement of supplementary ITM with OTM; TR (total replacement) represents comparison of basal diets + supplementary ITM vs. basal diets + total replacement of supplementary ITM with OTM. ^2^ Hens used in these trials were at the point of laying/start of egg production, which is usually between 15 and 18 weeks of age.

**Table 2:** Trace mineral concentration supplemented in layer diets of treatment comparisons involving total replacement of inorganic trace minerals (ITM) with organic trace minerals (OTM; Bioplex Cu, Fe, Mn, and Zn).

Trace Mineral	ITM Treatments (mg/kg)				OTM Treatments (mg/kg)			
	Mean	Minimum	Maximum	SD	Mean	Minimum	Maximum	SD
Zn	77.7	12.5	140.0	38.3	53.9	10.0	140.0	44.4
Mn	61.4	15.0	125.0	29.9	45.8	10.0	125.0	29.2
Cu	38.4	1.3	250.0	73.0	27.1	0.6	150.0	50.9
Fe	70.6	11.3	450.0	104.2	45.0	2.5	450.0	108.6

SD: standard deviation.

**Table 3:** Descriptive statistics of productive performance and egg quality traits included in the meta-analysis.

Item	*N*	Mean	Minimum	Maximum	SD
**Productive performance**					
Feed intake (g/day/hen)	71	114.91	80.43	145.00	12.09
Hen-day production (%)	92	84.22	66.29	98.11	7.09
FCR (g feed/kg egg)	63	2117.48	1480.00	4300.00	509.13
FCR (g feed/dozen eggs)	42	1813.21	1120.0	3450.00	544.74
**Egg quality**					
Egg mass (g/hen/day)	45	57.59	45.46	63.77	4.46
Egg weight (g)	87	64.71	56.51	68.91	3.06
Egg loss (%)	49	1.27	0.22	4.73	0.91
Eggshell thickness (mm)	43	0.35	0.11	0.50	0.08
Eggshell strength (kgf)	58	3.72	2.53	4.49	0.52
Eggshell weight (g)	41	8.45	5.47	9.77	1.65
Eggshell percentage (%)	43	8.60	6.81	14.60	1.15
Haugh unit	40	79.20	39.27	98.52	12.10

*N*: number of comparisons; SD: standard deviation.

**Table 4:** Overall pooled effect size of productive performance and egg quality traits when dietary organic trace minerals (Bioplex Cu, Fe, Mn, and Zn) were supplemented either in basal diets, partial replacement for inorganic trace minerals (ITM), or total replacement for ITM.

Item			Effect Size Estimates			Heterogeneity Tests	
	*N*	Control Mean (SD)	RMD (95% CI)	SE	*p*-Value	*I*^2^ (%)	*p*-Value
**Productive performance**							
Feed intake (g/day/hen)	71	115.06 (11.45)	−0.66 (−2.23, 0.91)	0.80	0.412	99.98	<0.001
Hen-day production (%)	92	83.63 (7.91)	2.07 (1.83, 2.31)	0.12	<0.001	99.67	<0.001
FCR (g feed/kg egg)	63	2143.73 (606.45)	−51.28 (−75.39, −27.18)	12.30	<0.001	98.50	<0.001
FCR (g feed/dozen eggs)	42	1869.21 (570.15)	−22.82 (−39.42, −6.23)	8.47	0.007	80.89	<0.001
**Egg quality**							
Egg mass (g/hen/day)	45	57.13 (4.41)	0.50 (0.13, 0.88)	0.19	0.008	79.25	<0.001
Egg weight (g)	87	64.41 (2.73)	0.48 (0.20, 0.77)	0.15	0.001	89.29	<0.001
Egg loss (%)	49	1.58 (1.04)	−0.62 (−0.73, −0.51)	0.06	<0.001	98.65	<0.001
Eggshell thickness (mm)	43	0.34 (0.08)	0.01 (0.01, 0.02)	0.00	<0.001	97.06	<0.001
Eggshell strength (kgf)	58	3.65 (0.49)	0.14 (0.07, 0.21)	0.04	<0.001	94.28	<0.001
Eggshell weight (g)	41	8.37 (1.71)	0.20 (0.12, 0.27)	0.04	<0.001	83.36	<0.001
Eggshell percentage (%)	43	8.53 (1.09)	0.15 (0.07, 0.22)	0.04	<0.001	95.58	<0.001
Haugh unit	40	78.86 (12.75)	0.89 (0.49, 1.29)	0.20	<0.001	89.73	<0.001

*N*: number of comparisons; SD standard deviation; RMD: raw mean difference and its associated 95% confidence interval; SE: standard error. *I*^2^: percentage of variation and associated significance level (*p*-value) of chi-squared statistic.

**Table 5:** Effect size of productive performance and egg quality traits when dietary organic trace minerals (Bioplex Cu, Fe, Mn, and Zn) were supplemented in basal diets.

Item			Effect Size Estimates			Heterogeneity Tests	
	*N*	Control Mean (SD)	RMD (95% CI)	SE	*p*-Value	*I*^2^ (%)	*p*-Value
**Productive performance**							
Feed intake (g/day/hen)	17	108.12 (13.59)	−2.12 (−5.66, 1.42)	1.81	0.241	99.21	<0.001
Hen-day production (%)	28	79.63 (7.82)	3.09 (0.55, 5.63)	1.30	0.017	99.19	<0.001
FCR (g feed/kg egg)	20	2011.40 (600.67)	−9.82 (−67.66, 48.03)	29.51	0.739	94.85	<0.001
FCR (g feed/dozen eggs)	14	1811.21 (539.64)	−62.68 (−126.21, 0.84)	32.41	0.053	83.55	<0.001
**Egg quality**							
Egg mass (g/hen/day)	9	53.50 (4.67)	0.69 (−0.35, 1.74)	0.53	0.193	86.20	<0.001
Egg weight (g)	25	63.52 (3.42)	0.25 (−0.45, 0.94)	0.35	0.486	93.37	<0.001
Egg loss (%)	9	2.13 (1.26)	−1.07 (−1.56, −0.59)	0.25	<0.001	99.10	<0.001
Eggshell thickness (mm)	16	0.35 (0.08)	0.02 (0.01, 0.03)	0.01	<0.001	98.62	<0.001
Eggshell strength (kgf)	10	3.38 (0.31)	0.11 (−0.09, 0.32)	0.10	0.272	93.35	<0.001
Eggshell weight (g)	5	6.89 (1.71)	0.69 (0.29, 1.10)	0.21	0.001	96.12	<0.001
Eggshell percentage (%)	17	8.58 (1.43)	0.27 (0.09, 0.44)	0.09	0.003	97.00	<0.001
Haugh unit	17	81.77 (10.12)	1.35 (0.52, 2.17)	0.42	0.001	79.43	<0.001

*N*: number of comparisons; SD standard deviation; RMD: raw mean difference and its associated 95% confidence interval; SE: standard error; *I*^2^: percentage of variation and associated significance level (*p*-value) of chi-squared statistic.

**Table 6:** Effect size of productive performance and egg quality traits when dietary organic trace minerals (Bioplex Cu, Fe, Mn, and Zn) were supplemented as total replacement for inorganic trace minerals.

Item			Effect Size Estimates			Heterogeneity Tests	
	*N*	Control Mean (SD)	RMD (95% CI)	SE	*p*-Value	*I*^2^ (%)	*p*-Value
**Productive performance**							
Feed intake (g/day/hen)	51	116.76 (9.93)	−0.05 (−1.91, 1.82)	0.95	0.963	99.99	<0.001
Hen-day production (%)	57	85.06 (7.62)	1.52 (1.16, 1.88)	0.18	<0.001	99.71	<0.001
FCR (g feed/kg egg)	41	2201.44 (620.89)	−73.09 (−107.24, −38.94)	17.42	<0.001	98.82	<0.001
FCR (g feed/dozen eggs)	25	1865.60 (619.92)	−19.32 (−37.14, −1.49)	9.10	0.034	81.12	<0.001
**Egg quality**							
Egg mass (g/hen/day)	36	58.04 (3.91)	0.50 (0.09, 0.90)	0.21	0.017	76.07	<0.001
Egg weight (g)	53	64.81 (2.47)	0.76 (0.43, 1.08)	0.17	<0.001	84.82	<0.001
Egg loss (%)	33	1.38 (0.91)	−0.57 (−0.75, −0.40)	0.09	<0.001	97.42	<0.001
Eggshell thickness (mm)	21	0.32 (0.09)	0.01 (0.01, 0.02)	0.00	<0.001	92.77	<0.001
Eggshell strength (kgf)	43	3.76 (0.51)	0.15 (0.07, 0.24)	0.04	<0.001	94.92	<0.001
Eggshell weight (g)	32	8.89 (1.43)	0.11 (0.06, 0.16)	0.03	<0.001	4.34	0.397
Eggshell percentage (%)	25	8.46 (0.82)	0.07 (−0.01, 0.143)	0.04	0.070	93.81	<0.001
Haugh unit	23	76.70 (14.22)	0.52 (0.10, 0.95)	0.22	0.016	88.60	<0.001

*N*: number of comparisons; SD standard deviation; RMD: raw mean difference and its associated 95% confidence interval; SE: standard error; *I*^2^: percentage of variation and associated significance level (*p*-value) of chi-squared statistic.

**Table 7:** Meta-regression of the effects of study factors on the overall pooled effect size of productive performance (feed intake, hen-day production, and feed conversion ratio) in response to supplementary organic trace minerals (Bioplex Cu, Fe, Mn, and Zn).

Study Factors	Feed Intake (g/day/hen)				Hen-Day Production (%)				Feed Conversion Ratio (g feed/kg egg)			
	Coefficient	SE	*p*-Value	*R*^2^ (%)	Coefficient	SE	*p*-Value	*R*^2^ (%)	Coefficient	SE	*p*-Value	*R*^2^ (%)
**Location**												
Africa	−15.21	2.31	<0.001	1.0	7.40	0.41	<0.001	17.0	−731.00	38.45	<0.001	41.0
Europe	−3.56	2.60	0.171		−0.68	0.39	0.082		32.60	68.55	0.634	
North America	−2.23	2.79	0.424		−0.55	0.45	0.225		62.84	29.40	0.033	
Oceania	-	-	-		-	-	-		−46.40	73.50	0.528	
South America	−2.01	2.14	0.347		−0.71	0.38	0.063		−30.53	23.74	0.199	
Asia	Referent ^1^				Referent ^1^				Referent ^1^			
**Year of study**	0.28	0.21	0.170	1.0	−0.18	0.02	<0.001	2.0	0.35	2.92	0.904	0.0
**Hen breed/strain**												
Black Harco	−15.74	3.61	<0.001	2.0	9.24	0.43	<0.001	18.0	-	-	-	56.0
Bovans	3.44	5.62	0.541		4.11	0.76	<0.001		209.35	67.27	0.002	
Brown Yaffa	−8.61	3.81	0.024		6.42	0.78	<0.001		−637.93	56.83	<0.001	
H&N Brown Nick	3.20	2.94	0.277		−1.04	0.68	0.126		98.33	50.13	0.050	
Hisex Brown	4.55	4.46	0.308		1.47	0.84	0.080		-	-	-	
Hy-Line	−0.49	2.89	0.865		0.86	0.37	0.022		100.25	48.99	0.041	
Isa Brown	-	-	-		1.56	0.45	<0.001		−30.68	58.20	0.598	
Roso SL hybrid	−0.47	6.84	0.946		5.93	1.28	<0.001		-	-	-	
White Leghorn	-	-	-		0.48	0.72	0.501		-	-	-	
Lohmann	Referent ^1^				Referent ^1^				Referent ^1^			
**Age of hens**	0.16	0.06	0.004	1.0	−0.05	0.01	<0.001	1.0	4.85	0.91	<0.001	0.0
**Number of hens**	0.00	0.00	0.864	0.0	−0.00	0.00	<0.001	0.0	−0.01	0.02	0.700	0.0
**Study duration**	0.03	0.05	0.555	1.0	−0.05	0.01	<0.001	1.0	6.24	1.39	<0.001	0.0

SE: standard error; *R*^2^: proportion of between-study variance (heterogeneity) explained by the study factors. ^1^ Study location and hen breed/strain were estimated by using Europe and Lohmann as the baseline, respectively.

**Table 8:** Meta-regression of the effects of study factors on the overall pooled effect size of egg quality traits (egg weight, egg loss and eggshell strength) in response to supplementary organic trace minerals (Bioplex Cu, Fe, Mn, and Zn).

Study Factors	Egg Weight (g)				Egg Loss (%)				Eggshell Strength (kgf)			
	Coefficient	SE	*p*-Value	*R*^2^ (%)	Coefficient	SE	*p*-Value	*R*^2^ (%)	Coefficient	SE	*p*-Value	*R*^2^ (%)
**Location**												
Africa	0.55	0.48	0.248	0.0	-	-	-	0.0	-	-	-	5.0
Europe	−0.74	0.46	0.110		−0.27	0.15	0.083		−0.13	0.11	0.225	
North America	−0.63	0.48	0.188		0.30	0.41	0.466		−0.34	0.09	<0.001	
Oceania	0.35	1.34	0.796		-	-	-		−0.40	0.25	0.106	
South America	−0.54	0.44	0.218		0.17	0.14	0.216		0.01	0.10	0.893	
Asia	Referent ^1^				Referent ^1^				Referent ^1^			
**Year of study**	−0.00	0.03	0.906	0.0	−0.02	0.01	0.141	0.0	−0.01	0.01	0.087	0.0
**Hen breed/strain**												
Black Harco	−0.29	0.51	0.574	44.0	-	-	-	20.0	-	-	-	10.0
Bovans	−2.14	0.62	0.001		-	-	-		-	-	-	
Brown Yaffa	2.89	0.56	<0.001		-	-	-		-	-	-	
H&N Brown Nick	1.11	0.48	0.021		1.19	0.22	<0.001		-	-	-	
Hy-Line	−0.24	0.42	0.565		1.40	0.23	<0.001		−0.19	0.08	0.012	
Isa Brown	0.36	0.48	0.455		1.37	0.25	<0.001		−0.21	0.11	0.046	
Jinghong	0.09	0.76	0.908		-	-	-		−0.34	0.13	0.010	
Roso SL hybrid	0.15	1.14	0.896		-	-	-		-	-	-	
White Leghorn	−0.00	0.62	0.998		-	-	-		-	-	-	
Lohmann	Referent ^1^				Referent ^1^				Referent ^1^			
**Age of hens**	−0.00	0.01	0.946	0.0	0.00	0.01	0.407	0.0	0.01	0.00	0.014	1.0
**Number of hens**	−0.00	0.00	0.520	0.0	0.00	0.00	0.027	8.0	−0.00	0.00	0.505	0.0
**Study duration**	−0.02	0.01	0.094	0.0	0.01	0.00	0.024	0.0	−0.01	0.00	0.002	4.0

SE: standard error; *R*^2^: proportion of between-study variance (heterogeneity) explained by the study factors. ^1^ Study location and hen breed/strain were estimated by using Europe and Lohmann as the baseline, respectively.

**Table 9:** Effect of supplementing organic trace minerals (OTM; Bioplex Cu, Fe, Mn, and Zn) on the carbon footprint of egg production.

	Low-GWP Diet			High-GWP Diet		
Category/Functional Unit	Baseline	OTM	% Change	Baseline	OTM	% Change
**Feed emission intensity**						
Emissions per dozen eggs (kg CO_2_-eq/dozen eggs)	1.27	1.25	−1.65%	1.58	1.56	−1.65%
Emissions per egg (g CO_2_-eq/egg)	105.86	104.10	−1.65%	131.80	129.70	−1.65%
Emissions per kg eggs (kg CO_2_-eq/kg eggs)	1.63	1.59	−2.40%	2.03	1.98	−2.40%
**Total emission intensity**						
Emissions per dozen eggs (kg CO_2_-eq/dozen eggs)	1.71	1.68	−1.75%	2.03	1.99	−1.73%
Emissions per egg (g CO_2_-eq/egg)	142.70	140.20	−1.75%	168.90	165.90	−1.73%
Emissions per kg eggs (kg CO_2_-eq/kg eggs)	2.19	2.14	−2.50%	2.60	2.53	−2.48%

GWP: global warming potential.

## Data Availability

All data and related tables generated during this meta-analysis are included in the published review and its Supplementary Information files. The review was not registered previously. The review protocol followed previous meta-analysis publications and was not prepared for external accession.

## Supplementary Materials

The following supporting information can be downloaded at: https://www.mdpi.com/article/10.3390/ani13193132/s1, Figure S1: Forest plot of the effect of organic trace mineral supplementation on feed intake (g/day/hen) of laying hens; Figure S2: Forest plot of the effect of organic trace mineral supplementation on hen-day production (%) of laying hens; Figure S3: Forest plot of the effect of organic trace mineral supplementation on FCR (g feed/kg egg) of laying hens; Figure S4: Forest plot of the effect of organic trace mineral supplementation on FCR (g feed/dozen egg) of laying hens; Figure S5: Forest plot of the effect of organic trace mineral supplementation on egg mass (g/hen/day) of laying hens; Figure S6: Forest plot of the effect of organic trace mineral supplementation on egg weight (g) of laying hens; Figure S7: Forest plot of the effect of organic trace mineral supplementation on egg loss (%) of laying hens; Figure S8: Forest plot of the effect of organic trace mineral supplementation on eggshell thickness (mm) of laying hens; Figure S9: Forest plot of the effect of organic trace mineral supplementation on eggshell strength (kgf) of laying hens; Figure S10: Forest plot of the effect of organic trace mineral supplementation on eggshell weight (g) of laying hens; Figure S11: Forest plot of the effect of organic trace mineral supplementation on eggshell percentage (%) of laying hens; Figure S12: Forest plot of the effect of organic trace mineral supplementation on Haugh unit of laying hens; Table S1: Data describing production characteristics and laying performance of baseline and organic trace mineral (OTM) scenarios; Table S2: Ingredient composition of formulated low- and high-soybean-meal (SBM) diets in different laying phases; Table S3: Summary of daily feed intake, crude protein content, and estimated global warming potential (GWP) of formulated low- and high-soybean-meal (SBM) diets.

## Abbreviations

CFP	Carbon Footprint
CMA	Comprehensive Meta-Analysis
Cu	Copper
CI	Confidence Interval
DEFRA	Department for Environment, Food and Rural Affairs
FCR	Feed Conversion Ratio
Fe	Iron
GHG	Greenhouse Gas
GWP	Global Warming Potential
HDP	Hen-Day Production
IPCC	Intergovernmental Panel on Climate Change
ITM	Inorganic Trace Minerals
LCA	Life Cycle Assessment
MD	Mean difference
Mn	Manganese
OTM	Organic Trace Minerals
PR	Partial Replacement
PRISMA	Preferred Reporting Items for Systematic Reviews and Meta-Analyses
RBV	Relative Bioavailability
RMD	Raw Mean Difference
ROI	Return On Investment
SBM	Soybean Meal
SD	Standard Deviation
SMD	Standardised Mean Difference
TR	Total Replacement
Zn	Zinc
