# Supplementary material for: The Effect of Organic Trace Mineral Supplementation in the Form of Proteinates on Performance and Sustainability Parameters in Laying Hens: A Meta-Analysis
Source: Animals (Basel). 2023 Oct 7;13(19):3132. doi: 10.3390/ani13193132 (PMC10571535; doi:10.3390/ani13193132)
Supplement: Supplementary file 1 [file animals-13-03132-s001.zip › OTM supplementary figures S1 to S12.pdf]

SUPPLEMENTARY FIGURES S1-S12

Studyname	Std diff in means	Lower limit	Upper limit	Relative weight
Murakami and Franco 2004	0,06	-0,15	0,28	1,63
Sara et al. 2008	0,00	-0,31	0,31	1,60
Schäublin et al. 2008	0,17	0,10	0,23	1,65
Klug et al. 2007 (1)	0,90	0,54	1,27	1,58
Klug et al. 2007 (2)	0,45	0,10	0,80	1,58
Klug et al. 2007 (3)	0,93	0,57	1,30	1,58
Güçlü et al. 2008 (1)	0,31	-0,35	0,97	1,42
Güçlü et al. 2008 (2)	0,50	-0,17	1,16	1,42
Güçlü et al. 2008 (3)	-0,16	-0,81	0,50	1,42
Ao et al. 2012 (1)	0,00	-0,28	0,28	1,61
Ao et al. 2012 (2)	0,00	-0,28	0,28	1,61
Stefanello et al. 2014 (1)	0,05	-0,39	0,49	1,54
Stefanello et al. 2014 (2)	0,04	-0,40	0,48	1,54
Stefanello et al. 2014 (3)	0,04	-0,40	0,48	1,54
Stefanello et al. 2014 (4)	0,01	-0,43	0,45	1,54
Stefanello et al. 2014 (5)	0,24	-0,20	0,68	1,54
Stefanello et al. 2014 (6)	-0,73	-1,18	-0,28	1,54
Stefanello et al. 2014 (7)	0,00	-0,44	0,44	1,54
Stefanello et al. 2014 (8)	0,00	-0,44	0,44	1,54
Ceylan and Scheideler 1999	0,92	0,43	1,40	1,52
Boruta et al. 2007 (1)	-0,19	-0,59	0,21	1,56
Boruta et al. 2007 (2)	-0,33	-0,74	0,07	1,56
Boruta et al. 2007 (3)	-0,24	-0,64	0,16	1,56
Ao et al. 2016 (1)	0,01	-0,29	0,31	1,60
Ao et al. 2016 (2)	-0,16	-0,46	0,14	1,60
Ao et al. 2016 (3)	-0,01	-0,31	0,29	1,60
Ao et al. 2018	-0,16	-0,41	0,10	1,62
Qiu et al. 2020 (1)	-0,07	-0,31	0,17	1,62
Qiu et al. 2020 (2)	-0,73	-0,98	-0,48	1,62
Yildiz et al. 2011 (1)	0,01	-0,71	0,72	1,39
Yildiz et al. 2011 (2)	0,35	-0,37	1,07	1,38
Yildiz et al. 2011 (3)	0,49	-0,23	1,22	1,38
Yildiz et al. 2011 (4)	-0,05	-0,76	0,67	1,39
Yildiz et al. 2011 (5)	1,18	0,40	1,96	1,35
Yildiz et al. 2011 (6)	0,71	-0,03	1,45	1,37
Yildiz et al. 2011 (7)	0,63	-0,10	1,36	1,37
Yildiz et al. 2011 (8)	0,12	-0,59	0,84	1,39
Yildiz et al. 2011 (9)	-0,17	-0,89	0,55	1,38
Yildiz et al. 2011 (10)	-0,25	-0,97	0,47	1,38
Yildiz et al. 2011 (11)	0,28	-0,44	1,00	1,38
Yildiz et al. 2011 (12)	-0,14	-0,86	0,57	1,39
Yildiz et al. 2011 (13)	-0,46	-1,19	0,26	1,38
Yildiz et al. 2011 (14)	0,10	-0,62	0,82	1,39
Yildiz et al. 2011 (15)	0,54	-0,19	1,27	1,38
Yildiz et al. 2011 (16)	0,18	-0,54	0,90	1,38
Yildiz et al. 2011 (17)	-0,01	-0,72	0,71	1,39
Yildiz et al. 2011 (18)	-0,14	-0,86	0,57	1,39
Yildiz et al. 2011 (19)	0,38	-0,34	1,10	1,38
Yildiz et al. 2011 (20)	0,32	-0,40	1,04	1,38
Yildiz et al. 2011 (21)	-0,31	-1,03	0,41	1,38
Yildiz et al. 2011 (22)	0,67	-0,07	1,40	1,37
Yildiz et al. 2011 (23)	3,00	1,96	4,05	1,17
Yildiz et al. 2011 (24)	2,79	1,79	3,80	1,19
Yildiz et al. 2011 (25)	1,41	0,61	2,21	1,33
Idowu et al. 2011 (1)	-2,08	-2,52	-1,63	1,54
Idowu et al. 2011 (2)	-0,78	-1,15	-0,41	1,57
Idowu et al. 2011 (3)	-16,67	-18,81	-14,53	0,60
Idowu et al. 2011 (4)	-200,00	-225,31	-174,69	0,01
Idowu et al. 2011 (5)	-1,04	-1,42	-0,66	1,57
Idowu et al. 2006 (1)	-2,57	-3,26	-1,89	1,41
Idowu et al. 2006 (2)	-11,18	-13,24	-9,11	0,63
Idowu et al. 2006 (3)	-1,82	-2,42	-1,21	1,46
Idowu et al. 2006 (4)	-10,89	-12,91	-8,88	0,65
Idowu et al. 2006 (5)	-11,56	-13,69	-9,43	0,61
Idowu et al. 2006 (6)	-0,94	-1,48	-0,41	1,49
Venglovská 2014 (1)	0,27	-0,29	0,84	1,47
Venglovská 2014 (2)	0,08	-0,49	0,64	1,48
Venglovská 2104 (3)	0,08	-0,49	0,64	1,48
Mehran Torki 2014	0,42	-0,09	0,93	1,51
Jim et al. 2018 (1)	-0,26	-0,61	0,09	1,58
Jim et al. 2018 (2)	0,22	-0,13	0,57	1,58
	-0,28	-0,48	-0,07	

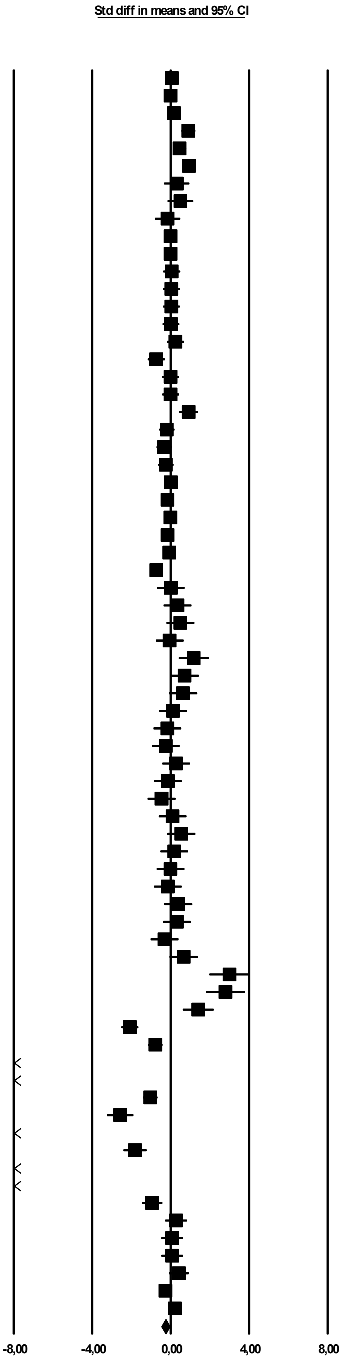

Figure S1: Forest plot of the effect of organic trace mineral supplementation on feed intake (g/day/hen) of laying hens. Study name refers to the reference of that study. Raw mean differences (difference in means) represent the effect size estimate. Each square represents

the mean effect size for that study. Thus, the squares to the right of the zero mid-line represent greater feed intake, squares to the left of the 0 mid-line indicate a lower feed intake and squares at the 0 mid-line indicate no effect on feed intake. The upper and lower limit of the line connected to the square represents the upper and lower 95% confidence interval (CI) for the effect size. The size of the square reflects the relative weighting of the study to the overall effect size estimate, with larger squares representing greater weight. The diamond at the bottom represents the overall effect size estimate.

Study name	Std diff in means	Lower limit	Upper limit	Relative weight
	Statistics for each study			
Sara et al. 2008	0,83	0,50	1,16	1,30
Schäublin et al. 2008	-0,03	-0,09	0,04	1,47
Klug et al. 2007 (1)	0,24	-0,11	0,58	1,28
Klug et al. 2007 (2)	0,00	-0,34	0,35	1,28
Klug et al. 2007 (3)	0,27	-0,08	0,62	1,28
Macisaac et al. 2006 (1)	-0,04	-0,37	0,28	1,30
Macisaac et al. 2006 (2)	0,28	-0,05	0,61	1,30
Macisaac et al. 2006 (3)	0,36	0,03	0,69	1,30
Macisaac et al. 2006 (4)	-0,51	-0,84	-0,17	1,29
Güçlü et al. 2008 (1)	1,72	0,95	2,48	0,84
Güçlü et al. 2008 (2)	2,32	1,47	3,16	0,76
Güçlü et al. 2008 (3)	-0,80	-1,48	-0,13	0,92
Leeson et al. 2010	-0,12	-0,27	0,02	1,44
Sefton and Leeson 2011	-0,20	-0,66	0,26	1,15
Ao et al. 2012 (1)	-0,01	-0,29	0,28	1,34
Ao et al. 2012 (2)	0,02	-0,27	0,30	1,34
Stefanello et al. 2014 (1)	-0,01	-0,45	0,42	1,18
Stefanello et al. 2014 (2)	0,12	-0,32	0,56	1,18
Stefanello et al. 2014 (3)	0,13	-0,31	0,57	1,18
Stefanello et al. 2014 (4)	0,09	-0,35	0,53	1,18
Stefanello et al. 2014 (5)	-0,73	-1,18	-0,27	1,17
Stefanello et al. 2014 (6)	-0,09	-0,53	0,35	1,18
Stefanello et al. 2014 (7)	0,45	0,01	0,90	1,18
Stefanello et al. 2014 (8)	-0,09	-0,53	0,35	1,18
Nolan 2019 (1)	1,34	0,89	1,78	1,18
Nolan 2019 (2)	1,21	0,78	1,65	1,19
Nolan 2019 (3)	0,00	-0,40	0,40	1,22
Nolan 2019 (4)	-0,21	-0,61	0,19	1,22
Nolan 2019 (5)	-0,17	-0,57	0,23	1,22
Nolan 2019 (6)	-0,32	-0,72	0,08	1,22
Nolan 2019 (7)	0,42	0,02	0,83	1,22
Nolan 2019 (8)	-0,02	-0,42	0,38	1,22
Ceylan and Scheideler 1999	0,09	-0,38	0,55	1,16
Carlos and Solano 2005 (trial 1)	0,16	0,11	0,20	1,48
Carlos and Solano 2005 (trial 2)	0,09	0,04	0,14	1,48
Siske 2000 (1.1)	0,64	0,45	0,84	1,41
Siske 2000 (1.2)	0,47	0,28	0,66	1,41
Siske 2000 (2.1)	0,05	-0,17	0,27	1,39
Siske 2000 (2.2)	-0,20	-0,42	0,02	1,39
Siske 2000 (2.3)	0,20	-0,02	0,42	1,39
Siske 2000 (3)	0,14	-0,02	0,29	1,44
Xavier et al. 2004 (1)	-0,20	-0,55	0,14	1,28
Xavier et al. 2004 (2)	0,60	0,25	0,96	1,27
Xavier et al. 2004 (3)	0,77	0,41	1,12	1,27
Ao et al. 2016 (1)	-0,07	-0,37	0,24	1,32
Ao et al. 2016 (2)	0,02	-0,28	0,32	1,32
Ao et al. 2016 (3)	-0,10	-0,41	0,20	1,32
Ao et al. 2018	0,08	-0,17	0,34	1,37
Qiu et al. 2020 (1)	-0,45	-0,69	-0,21	1,38
Qiu et al. 2020 (2)	2,41	2,09	2,72	1,31
Yildiz et al. 2011 (1)	-0,14	-0,86	0,58	0,88
Yildiz et al. 2011 (2)	-0,15	-0,87	0,57	0,88
Yildiz et al. 2011 (3)	-0,24	-0,96	0,47	0,88
Yildiz et al. 2011 (4)	-0,44	-1,16	0,29	0,87
Yildiz et al. 2011 (5)	0,59	-0,14	1,32	0,87
Yildiz et al. 2011 (6)	-0,14	-0,86	0,57	0,88
Yildiz et al. 2011 (7)	-0,43	-1,15	0,30	0,88
Yildiz et al. 2011 (8)	-0,24	-0,96	0,48	0,88
Yildiz et al. 2011 (9)	0,03	-0,69	0,75	0,88
Yildiz et al. 2011 (10)	-0,15	-0,87	0,57	0,88
Yildiz et al. 2011 (11)	-0,23	-0,95	0,48	0,88
Yildiz et al. 2011 (12)	-0,24	-0,96	0,47	0,88
Yildiz et al. 2011 (13)	0,01	-0,71	0,73	0,88
Yildiz et al. 2011 (14)	-0,18	-0,90	0,54	0,88
Yildiz et al. 2011 (15)	-0,18	-0,89	0,54	0,88
Yildiz et al. 2011 (16)	-0,26	-0,97	0,46	0,88
Yildiz et al. 2011 (17)	-0,05	-0,77	0,67	0,88
Yildiz et al. 2011 (18)	-0,31	-1,03	0,41	0,88
Yildiz et al. 2011 (19)	-0,30	-1,02	0,42	0,88
Yildiz et al. 2011 (20)	-0,33	-1,05	0,39	0,88
Yildiz et al. 2011 (21)	-0,17	-0,88	0,55	0,88
Yildiz et al. 2011 (22)	-0,15	-0,86	0,57	0,88
Yildiz et al. 2011 (23)	-0,15	-0,87	0,57	0,88
Yildiz et al. 2011 (24)	-0,23	-0,95	0,49	0,88
Yildiz et al. 2011 (25)	-0,23	-0,95	0,48	0,88
Idowu et al. 2011 (1)	0,74	0,37	1,11	1,26
Idowu et al. 2011 (2)	0,86	0,49	1,23	1,25
Idowu et al. 2011 (3)	0,34	-0,03	0,70	1,27
Idowu et al. 2011 (4)	0,95	0,57	1,32	1,25
Idowu et al. 2011 (5)	0,94	0,56	1,31	1,25
Idowu et al. 2006 (1)	9,60	7,81	11,39	0,28
Idowu et al. 2006 (2)	10,55	8,60	12,51	0,24
Idowu et al. 2006 (3)	9,13	7,42	10,84	0,30
Idowu et al. 2006 (4)	3,39	2,60	4,18	0,81
Idowu et al. 2006 (5)	10,10	8,23	11,98	0,26
Idowu et al. 2006 (6)	-1,92	-2,53	-1,31	0,99
Venglovská 2014 (1)	0,00	-0,57	0,57	1,04
Venglovská 2014 (2)	0,00	-0,57	0,57	1,04
Venglovská 2104 (3)	-50,00	-60,02	-39,98	0,01
Mehran Torki 2014	1,20	0,65	1,75	1,06
Jim et al. 2018 (1)	-0,07	-0,42	0,27	1,28
Jim et al. 2018 (2)	-0,27	-0,62	0,08	1,28
	0,25	0,15	0,36	

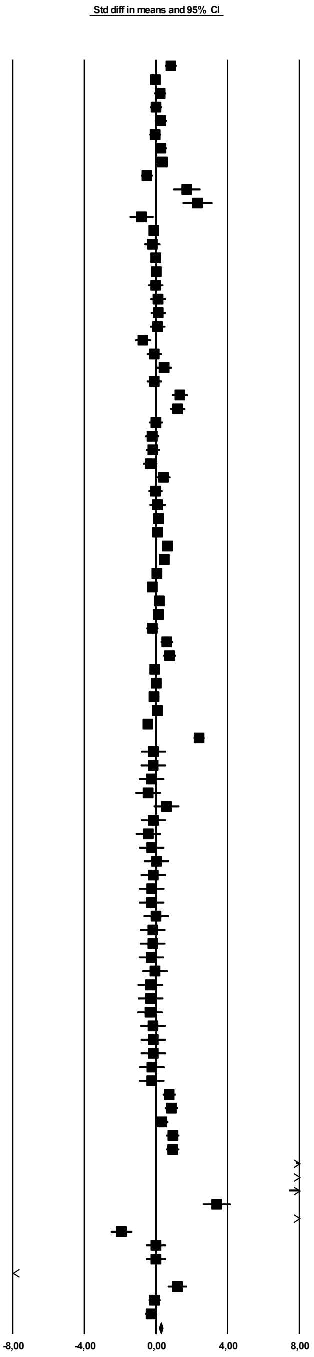

Figure S2: Forest plot of the effect of organic trace mineral supplementation on hen-day production (%) of laying hens. Study name refers to the reference of that study. Raw mean differences (difference in means) represent the effect size estimate. Each square represents the mean effect size for that study. Thus, the squares to the right of the zero mid-line represent higher hen-day production, squares to the left of the 0 mid-line indicate lower hen-day production and squares at the 0 mid-line indicate no effect on hen-day production. The

upper and lower limit of the line connected to the square represents the upper and lower 95% confidence interval (CI) for the effect size. The size of the square reflects the relative weighting of the study to the overall effect size estimate, with larger squares representing greater weight. The diamond at the bottom represents the overall effect size estimate.

Study name	Std diff in means	Lower limit	Upper limit	Relative weight
Murakami and Franco 2004	0,22	0,00	0,44	1,42
Schäublin et al. 2008	0,24	0,18	0,31	16,64
Güçlü et al. 2008 (1)	0,72	0,05	1,39	0,15
Güçlü et al. 2008 (2)	1,31	0,59	2,03	0,13
Güçlü et al. 2008 (3)	0,14	-0,51	0,80	0,16
Stefanello et al. 2014 (1)	0,06	-0,38	0,50	0,36
Stefanello et al. 2014 (2)	0,00	-0,44	0,44	0,36
Stefanello et al. 2014 (3)	0,00	-0,44	0,44	0,36
Stefanello et al. 2014 (4)	-0,06	-0,50	0,38	0,36
Stefanello et al. 2014 (5)	0,29	-0,15	0,73	0,35
Stefanello et al. 2014 (6)	0,29	-0,15	0,73	0,35
Stefanello et al. 2014 (7)	0,00	-0,44	0,44	0,36
Stefanello et al. 2014 (8)	0,00	-0,44	0,44	0,36
Ndlan 2019 (1)	0,25	-0,15	0,65	0,43
Ndlan 2019 (2)	0,06	-0,34	0,46	0,43
Ndlan 2019 (3)	0,25	-0,15	0,65	0,43
Ndlan 2019 (4)	0,00	-0,40	0,40	0,43
Ndlan 2019 (5)	0,44	0,03	0,84	0,42
Ndlan 2019 (6)	-0,25	-0,65	0,15	0,43
Ndlan 2019 (7)	0,44	0,03	0,84	0,42
Ndlan 2019 (8)	-0,31	-0,72	0,09	0,42
Ceylan and Scheideler 1999	3,19	2,49	3,88	0,14
Carlos and Solano 2005 (trial 1)	-0,24	-0,29	-0,20	35,55
Carlos and Solano 2005 (trial 2)	-0,63	-0,67	-0,58	30,09
Xavier et al. 2004 (1)	-0,28	-0,62	0,07	0,57
Xavier et al. 2004 (2)	-0,80	-1,16	-0,44	0,53
Xavier et al. 2004 (3)	-0,95	-1,32	-0,58	0,51
Qiu et al. 2020 (1)	-0,67	-0,91	-0,42	1,14
Qiu et al. 2020 (2)	-4,40	-4,84	-3,96	0,35
Yildiz et al. 2011 (1)	-0,14	-0,86	0,58	0,13
Yildiz et al. 2011 (2)	-0,14	-0,86	0,57	0,13
Yildiz et al. 2011 (3)	0,31	-0,41	1,03	0,13
Yildiz et al. 2011 (4)	0,26	-0,46	0,98	0,13
Yildiz et al. 2011 (5)	-0,09	-0,81	0,62	0,13
Yildiz et al. 2011 (6)	0,43	-0,30	1,15	0,13
Yildiz et al. 2011 (7)	0,57	-0,16	1,30	0,13
Yildiz et al. 2011 (8)	0,55	-0,18	1,27	0,13
Yildiz et al. 2011 (9)	-0,30	-1,02	0,42	0,13
Yildiz et al. 2011 (10)	-0,48	-1,21	0,24	0,13
Yildiz et al. 2011 (11)	0,02	-0,69	0,74	0,13
Yildiz et al. 2011 (12)	-0,13	-0,85	0,58	0,13
Yildiz et al. 2011 (13)	-0,65	-1,39	0,08	0,13
Yildiz et al. 2011 (14)	-0,15	-0,86	0,57	0,13
Yildiz et al. 2011 (15)	0,20	-0,52	0,92	0,13
Yildiz et al. 2011 (16)	0,24	-0,47	0,96	0,13
Yildiz et al. 2011 (17)	-0,24	-0,96	0,47	0,13
Yildiz et al. 2011 (18)	-0,15	-0,87	0,56	0,13
Yildiz et al. 2011 (19)	0,21	-0,50	0,93	0,13
Yildiz et al. 2011 (20)	0,33	-0,39	1,05	0,13
Yildiz et al. 2011 (21)	-0,26	-0,98	0,46	0,13
Yildiz et al. 2011 (22)	0,01	-0,70	0,73	0,13
Yildiz et al. 2011 (23)	0,52	-0,20	1,25	0,13
Yildiz et al. 2011 (24)	0,63	-0,10	1,37	0,13
Yildiz et al. 2011 (25)	0,64	-0,10	1,37	0,13
Idowu et al. 2011 (1)	-1,56	-1,97	-1,15	0,41
Idowu et al. 2011 (2)	-5,63	-6,42	-4,83	0,11
Idowu et al. 2011 (3)	-2,88	-3,39	-2,36	0,26
Idowu et al. 2011 (4)	-5,00	-5,73	-4,27	0,13
Idowu et al. 2011 (5)	-4,13	-4,76	-3,49	0,17
Mehran Torki 2014	-1,30	-1,86	-0,74	0,22
Kocher et al. 2020	-0,25	-0,58	0,08	0,62
Jim et al. 2018 (1)	0,23	-0,11	0,58	0,57
Jim et al. 2018 (2)	0,59	0,24	0,95	0,55
	-0,28	-0,31	-0,26	

(Statistics for each study)

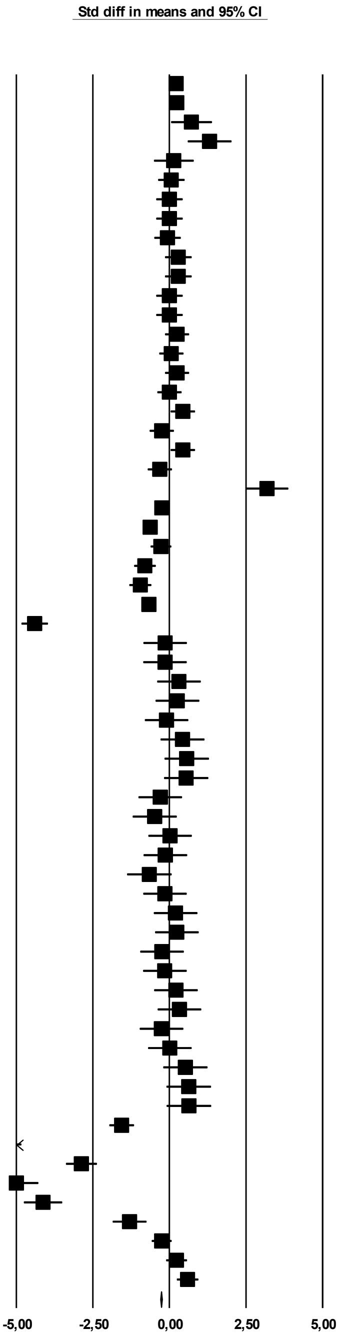

Figure S3: Forest plot of the effect of organic trace mineral supplementation on FCR (g feed/kg egg) of laying hens. Study name refers to the reference of that study. Raw mean differences (difference in means) represent the effect size estimate. Each square represents the mean effect size for that study. Thus, the squares to the right of the zero mid-line represent a higher FCR, squares to the left of the 0 mid-line indicate a lower FCR and squares at the 0 mid-line indicate no effect on FCR. The upper and lower limit of the line connected

to the square represents the upper and lower 95% confidence interval (CI) for the effect size. The size of the square reflects the relative weighting of the study to the overall effect size estimate, with larger squares representing greater weight. The diamond at the bottom represents the overall effect size estimate.

Study name	Std diff in means	Lower limit	Upper limit	Relative weight
Murakami and Franco 2004	-0,03	-0,25	0,18	2,92
Klug et al. 2007 (1)	0,34	-0,01	0,69	2,53
Klug et al. 2007 (2)	0,27	-0,08	0,62	2,54
Klug et al. 2007 (3)	0,34	-0,01	0,69	2,53
Ao et al. 2012 (1)	-0,04	-0,32	0,25	2,74
Ao et al. 2012 (2)	-0,07	-0,36	0,21	2,74
Stefanello et al. 2014 (1)	0,00	-0,44	0,44	2,25
Stefanello et al. 2014 (2)	-0,03	-0,47	0,40	2,25
Stefanello et al. 2014 (3)	0,00	-0,44	0,44	2,25
Stefanello et al. 2014 (4)	0,00	-0,44	0,44	2,25
Stefanello et al. 2014 (5)	0,00	-0,44	0,44	2,25
Stefanello et al. 2014 (6)	0,00	-0,44	0,44	2,25
Stefanello et al. 2014 (7)	0,45	0,01	0,90	2,23
Stefanello et al. 2014 (8)	0,00	-0,44	0,44	2,25
Nolan 2019 (1)	-0,10	-0,50	0,30	2,37
Nolan 2019 (2)	0,24	-0,16	0,64	2,37
Nolan 2019 (3)	0,03	-0,37	0,43	2,37
Nolan 2019 (4)	-0,10	-0,50	0,30	2,37
Nolan 2019 (5)	0,10	-0,30	0,50	2,37
Nolan 2019 (6)	0,34	-0,07	0,74	2,36
Nolan 2019 (7)	-0,10	-0,50	0,30	2,37
Nolan 2019 (8)	-0,14	-0,54	0,27	2,37
Boruta et al. 2007 (1)	-0,20	-0,60	0,20	2,37
Boruta et al. 2007 (2)	-0,41	-0,81	-0,00	2,36
Boruta et al. 2007 (3)	-0,14	-0,54	0,27	2,37
Ao et al. 2016 (1)	0,04	-0,26	0,34	2,68
Ao et al. 2016 (2)	-0,12	-0,42	0,19	2,68
Ao et al. 2016 (3)	0,08	-0,23	0,38	2,68
Ao et al. 2018	-0,20	-0,46	0,05	2,82
Idowu et al. 2011 (1)	-0,83	-1,20	-0,45	2,46
Idowu et al. 2011 (2)	-1,19	-1,58	-0,80	2,41
Idowu et al. 2011 (3)	-0,29	-0,65	0,07	2,50
Idowu et al. 2011 (4)	-0,58	-0,94	-0,21	2,48
Idowu et al. 2011 (5)	-0,57	-0,94	-0,21	2,48
Idowu et al. 2006 (1)	-0,88	-1,41	-0,35	1,97
Idowu et al. 2006 (2)	-1,64	-2,22	-1,05	1,82
Idowu et al. 2006 (3)	-0,48	-1,00	0,03	2,02
Idowu et al. 2006 (4)	-1,01	-1,55	-0,47	1,95
Idowu et al. 2006 (5)	-1,27	-1,83	-0,72	1,90
Idowu et al. 2006 (6)	-0,20	-0,70	0,31	2,04
Jim et al. 2018 (1)	-0,08	-0,43	0,26	2,54
Jim et al. 2018 (2)	0,39	0,04	0,74	2,53
	-0,17	-0,29	-0,05	

Statistics for each study

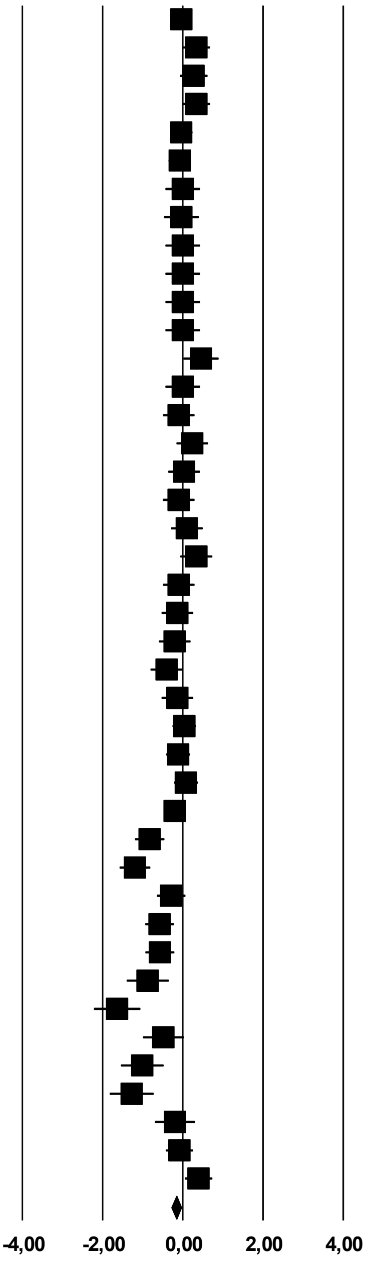

Figure S4: Forest plot of the effect of organic trace mineral supplementation on FCR (g feed/dozen egg) of laying hens. Study name refers to the reference of that study. Raw mean differences (difference in means) represent the effect size estimate. Each square represents the mean effect size for that study. Thus, the squares to the right of the zero mid-line represent a higher FCR, squares to the left of the 0 mid-line indicate a lower FCR and squares at the 0 mid-line indicate no effect on FCR. The upper and lower limit of the line connected

to the square represents the upper and lower 95% confidence interval (CI) for the effect size. The size of the square reflects the relative weighting of the study to the overall effect size estimate, with larger squares representing greater weight. The diamond at the bottom represents the overall effect size estimate.

Study name	Std diff in means	Lower limit	Upper limit	Relative weight
Yildiz et al. 2011 (18)	-0,01	-0,73	0,70	1,10
Yildiz et al. 2011 (16)	0,00	-0,71	0,72	1,10
Yildiz et al. 2011 (7)	-0,03	-0,74	0,69	1,10
Yildiz et al. 2011 (15)	0,34	-0,38	1,06	1,08
Yildiz et al. 2011 (22)	0,65	-0,08	1,38	1,04
Yildiz et al. 2011 (17)	0,21	-0,51	0,93	1,09
Yildiz et al. 2011 (11)	0,15	-0,57	0,86	1,09
Yildiz et al. 2011 (8)	-0,20	-0,91	0,52	1,09
Yildiz et al. 2011 (6)	0,19	-0,52	0,91	1,09
Yildiz et al. 2011 (24)	0,66	-0,07	1,40	1,04
Yildiz et al. 2011 (9)	0,04	-0,68	0,75	1,10
Yildiz et al. 2011 (14)	0,18	-0,54	0,89	1,09
Yildiz et al. 2011 (12)	-0,00	-0,72	0,71	1,10
Yildiz et al. 2011 (2)	0,41	-0,31	1,13	1,07
Yildiz et al. 2011 (13)	0,26	-0,46	0,98	1,09
Ceylan and Scheideler 1999	0,08	-0,38	0,54	2,63
Yildiz et al. 2011 (25)	0,60	-0,13	1,33	1,05
Yildiz et al. 2011 (23)	1,10	0,33	1,87	0,95
Yildiz et al. 2011 (5)	0,95	0,20	1,71	0,99
Mehran Torki 2014	1,51	0,94	2,08	1,71
Klug et al. 2007 (3)	0,05	-0,29	0,40	4,68
Klug et al. 2007 (2)	0,02	-0,33	0,36	4,68
Yildiz et al. 2011 (20)	-0,08	-0,80	0,64	1,10
Klug et al. 2007 (1)	-0,01	-0,35	0,34	4,68
Stefanello et al. 2014 (1)	0,40	-0,04	0,84	2,87
Stefanello et al. 2014 (2)	0,25	-0,19	0,69	2,90
Stefanello et al. 2014 (3)	0,36	-0,08	0,80	2,88
Stefanello et al. 2014 (4)	0,33	-0,12	0,77	2,89
Jim et al. 2018 (1)	-0,41	-0,76	-0,06	4,59
Jim et al. 2018 (2)	-0,45	-0,80	-0,10	4,57
Venglovská 2014 (1)	-0,47	-1,04	0,10	1,71
Venglovská 2014 (2)	-0,70	-1,28	-0,12	1,65
Venglovská 2104 (3)	0,02	-0,55	0,59	1,76
Yildiz et al. 2011 (3)	0,13	-0,59	0,84	1,10
Stefanello et al. 2014 (5)	0,96	0,50	1,43	2,62
Stefanello et al. 2014 (6)	-0,12	-0,56	0,32	2,92
Stefanello et al. 2014 (7)	0,24	-0,20	0,68	2,91
Stefanello et al. 2014 (8)	0,00	-0,44	0,44	2,93
Qiu et al. 2020 (1)	0,14	-0,10	0,38	9,86
Qiu et al. 2020 (2)	1,54	1,27	1,81	7,62
Yildiz et al. 2011 (1)	0,05	-0,67	0,76	1,10
Yildiz et al. 2011 (10)	0,20	-0,52	0,92	1,09
Yildiz et al. 2011 (4)	-0,22	-0,94	0,50	1,09
Yildiz et al. 2011 (19)	0,08	-0,63	0,80	1,10
Yildiz et al. 2011 (21)	-0,04	-0,76	0,68	1,10
	0,23	0,15	0,30	

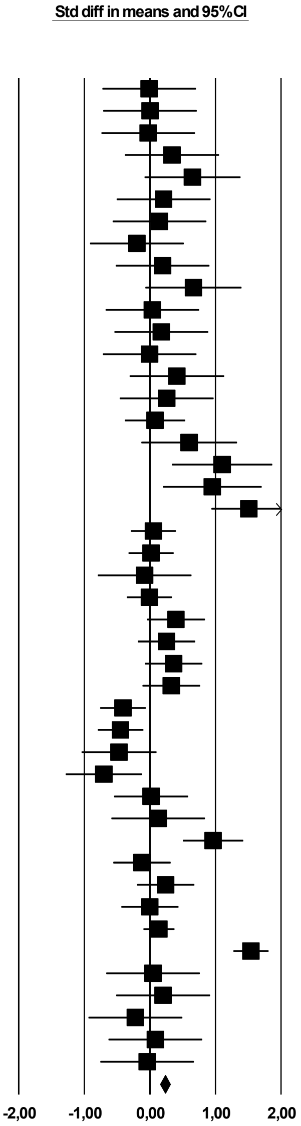

Figure S5: Forest plot of the effect of organic trace mineral supplementation on egg mass (g/hen/day) of laying hens. Study name refers to the reference of that study. Raw mean differences (difference in means) represent the effect size estimate. Each square represents the mean effect size for that study. Thus, the squares to the right of the zero mid-line represent higher egg mass, squares to the left of the 0 mid-line indicate lower egg mass and squares at the 0 mid-line indicate no effect on egg mass. The upper and lower limit of the line connected to the square represents the upper and lower 95% confidence interval (CI) for the effect size. The size of the square reflects the relative weighting of the study to the overall

effect size estimate, with larger squares representing greater weight. The diamond at the bottom represents the overall effect size estimate.

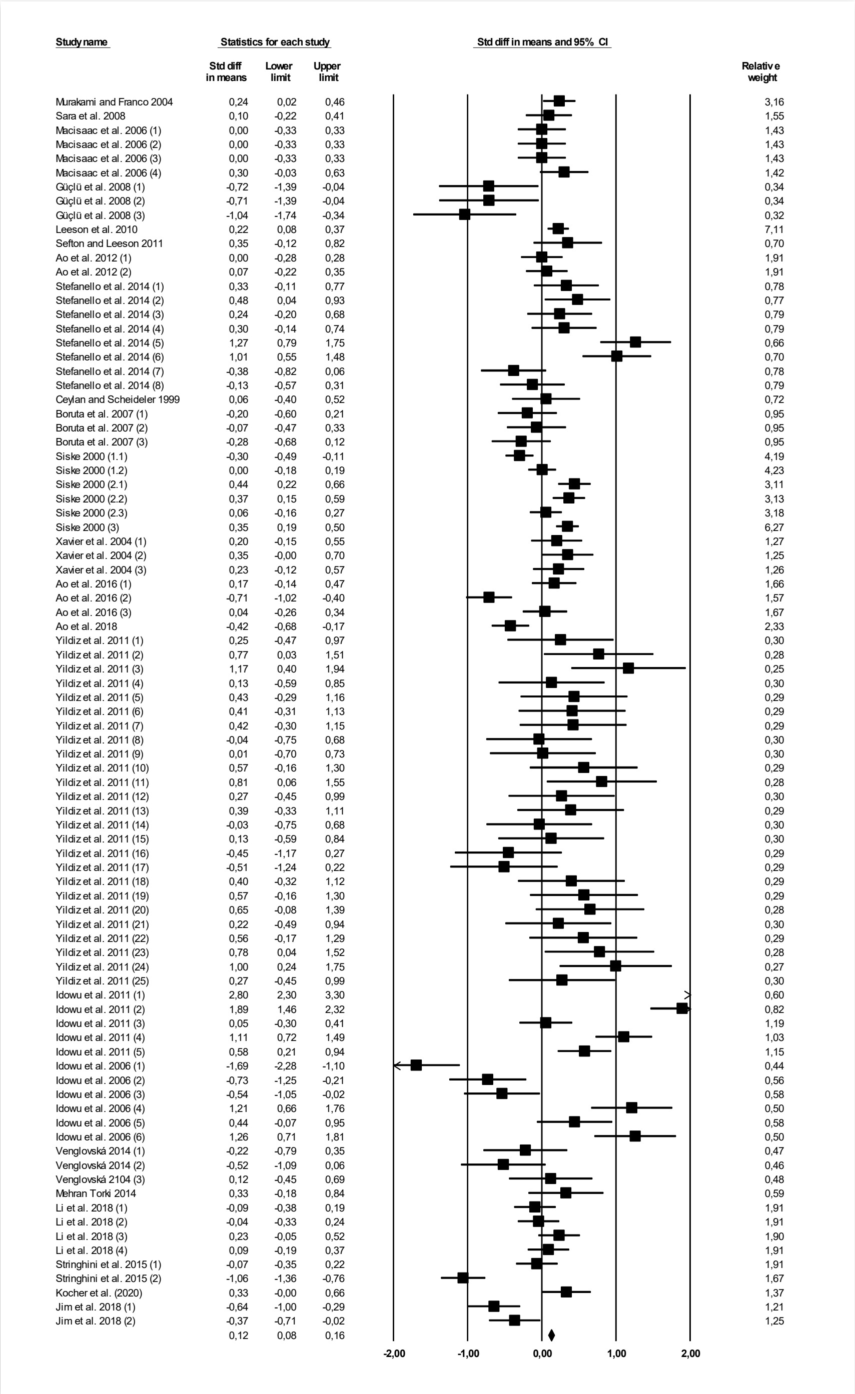

Figure S6: Forest plot of the effect of organic trace mineral supplementation on egg weight (g) of laying hens. Study name refers to the reference of that study. Raw mean differences (difference in means) represent the effect size estimate. Each square represents the mean effect size for that study. Thus, the squares to the right of the zero mid-line represent higher egg weight, squares to the left of the 0 mid-line indicate lower egg weight and squares at the

0 mid-line indicate no effect on egg weight. The upper and lower limit of the line connected to the square represents the upper and lower 95% confidence interval (CI) for the effect size. The size of the square reflects the relative weighting of the study to the overall effect size estimate, with larger squares representing greater weight. The diamond at the bottom represents the overall effect size estimate.

Study name	Statistics for each study			Relative weight
	Std diff in means	Lower limit	Upper limit	
Stefanello et al. 2014 (1)	-0,78	-1,24	-0,33	0,39
Stefanello et al. 2014 (2)	-1,70	-2,21	-1,18	0,31
Stefanello et al. 2014 (3)	-2,35	-2,92	-1,78	0,25
Stefanello et al. 2014 (4)	-2,93	-3,57	-2,30	0,20
Stefanello et al. 2014 (5)	-0,48	-0,92	-0,03	0,41
Stefanello et al. 2014 (6)	-0,42	-0,87	0,02	0,42
Stefanello et al. 2014 (7)	-0,14	-0,58	0,30	0,42
Stefanello et al. 2014 (8)	-0,14	-0,58	0,30	0,42
Ceylan and Scheideler 1999	-0,70	-1,17	-0,22	0,36
Carlos and Solano 2005 (trial 1)	-0,09	-0,13	-0,04	42,48
Carlos and Solano 2005 (trial 2)	-0,30	-0,35	-0,26	37,04
Siske 2000 (1.1)	-0,43	-0,63	-0,24	2,21
Siske 2000 (1.2)	-1,09	-1,29	-0,88	1,97
Siske 2000 (2.1)	-0,28	-0,50	-0,06	1,68
Siske 2000 (2.2)	-0,17	-0,39	0,05	1,69
Siske 2000 (2.3)	-1,91	-2,18	-1,65	1,17
Siske 2000 (3)	-2,04	-2,23	-1,85	2,23
Qiu et al. 2020 (1)	-1,23	-1,49	-0,97	1,21
Qiu et al. 2020 (2)	-4,00	-4,41	-3,59	0,48
Yildiz et al. 2011 (1)	-1,24	-2,02	-0,46	0,13
Yildiz et al. 2011 (2)	-5,11	-6,59	-3,63	0,04
Yildiz et al. 2011 (3)	-0,02	-0,74	0,69	0,16
Yildiz et al. 2011 (4)	-1,09	-1,85	-0,32	0,14
Yildiz et al. 2011 (5)	0,50	-0,23	1,23	0,15
Yildiz et al. 2011 (6)	-5,37	-6,91	-3,83	0,03
Yildiz et al. 2011 (7)	-0,09	-0,80	0,63	0,16
Yildiz et al. 2011 (8)	-1,28	-2,07	-0,50	0,13
Yildiz et al. 2011 (9)	0,61	-0,12	1,34	0,15
Yildiz et al. 2011 (10)	-0,98	-1,74	-0,22	0,14
Yildiz et al. 2011 (11)	0,17	-0,54	0,89	0,16
Yildiz et al. 2011 (12)	-1,02	-1,78	-0,26	0,14
Yildiz et al. 2011 (13)	0,87	0,12	1,62	0,15
Yildiz et al. 2011 (14)	-1,17	-1,95	-0,40	0,14
Yildiz et al. 2011 (15)	-5,30	-6,83	-3,78	0,04
Yildiz et al. 2011 (16)	-1,22	-2,00	-0,44	0,13
Yildiz et al. 2011 (17)	0,67	-0,06	1,41	0,15
Yildiz et al. 2011 (18)	-1,04	-1,81	-0,28	0,14
Yildiz et al. 2011 (19)	-5,17	-6,67	-3,68	0,04
Yildiz et al. 2011 (20)	0,11	-0,61	0,82	0,16
Yildiz et al. 2011 (21)	0,80	0,06	1,55	0,15
Yildiz et al. 2011 (22)	-1,35	-2,14	-0,56	0,13
Yildiz et al. 2011 (23)	-5,48	-7,04	-3,92	0,03
Yildiz et al. 2011 (24)	-0,20	-0,91	0,52	0,16
Yildiz et al. 2011 (25)	-1,39	-2,19	-0,59	0,13
Venglovská 2014 (1)	-9,07	-10,96	-7,17	0,02
Venglovská 2014 (2)	-0,78	-1,37	-0,20	0,24
Venglovská 2104 (3)	-2,09	-2,79	-1,38	0,17
Jim et al. 2018 (1)	-1,74	-2,15	-1,33	0,49
Jim et al. 2018 (2)	-0,83	-1,19	-0,47	0,63
	-0,36	-0,39	-0,33	

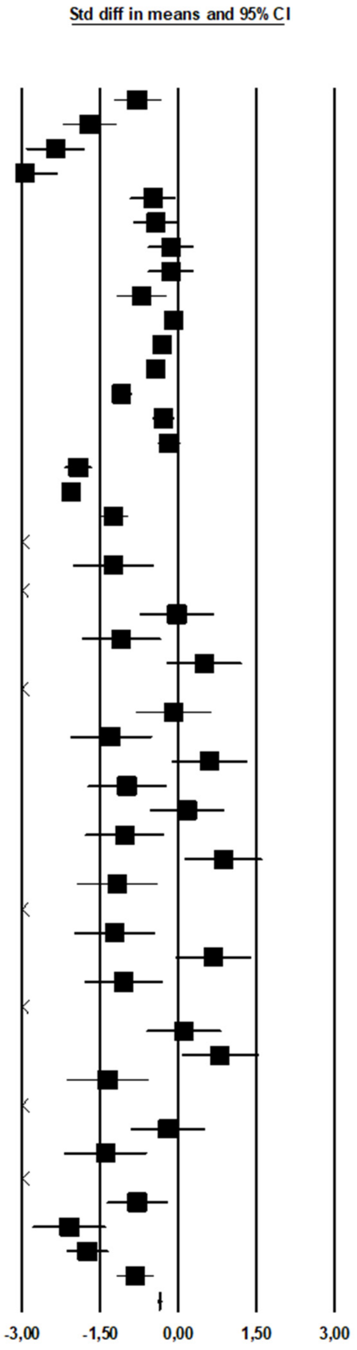

Figure S7: Forest plot of the effect of organic trace mineral supplementation on egg loss (%) of laying hens. Study name refers to the reference of that study. Raw mean differences (difference in means) represent the effect size estimate. Each square represents the mean effect size for that study. Thus, the squares to the right of the zero mid-line represent higher egg loss, squares to the left of the 0 mid-line indicate lower egg loss and squares at the 0 mid-line indicate no effect on egg loss. The upper and lower limit of the line connected to the

square represents the upper and lower 95% confidence interval (CI) for the effect size. The size of the square reflects the relative weighting of the study to the overall effect size estimate, with larger squares representing greater weight. The diamond at the bottom represents the overall effect size estimate.

Study name	Std diff in means	Lower limit	Upper limit	Relative weight
Murakami and Franco 2004	0,00	-0,22	0,22	2,51
Sara et al. 2008	5,00	4,36	5,64	2,04
Stefanello et al. 2014 (1)	0,50	0,05	0,95	2,29
Stefanello et al. 2014 (2)	1,00	0,54	1,46	2,27
Stefanello et al. 2014 (3)	1,50	1,00	2,00	2,23
Stefanello et al. 2014 (4)	1,50	1,00	2,00	2,23
Stefanello et al. 2014 (5)	0,00	-0,44	0,44	2,30
Stefanello et al. 2014 (6)	1,00	0,54	1,46	2,27
Stefanello et al. 2014 (7)	1,00	0,54	1,46	2,27
Stefanello et al. 2014 (8)	1,00	0,54	1,46	2,27
Siske 2000 (1.1)	0,00	-0,19	0,19	2,53
Siske 2000 (1.2)	0,50	0,31	0,69	2,53
Siske 2000 (2.1)	0,00	-0,22	0,22	2,51
Siske 2000 (2.2)	0,00	-0,22	0,22	2,51
Siske 2000 (2.3)	0,50	0,28	0,72	2,51
Siske 2000 (3)	0,50	0,34	0,66	2,55
Xavier et al. 2004 (1)	0,60	0,25	0,95	2,39
Xavier et al. 2004 (2)	0,80	0,44	1,16	2,39
Xavier et al. 2004 (3)	1,20	0,82	1,58	2,37
Qiu et al. 2020 (1)	0,00	-0,24	0,24	2,50
Qiu et al. 2020 (2)	0,33	0,09	0,57	2,50
Idowu et al. 2011 (1)	2,00	1,56	2,44	2,30
Idowu et al. 2011 (2)	1,50	1,09	1,91	2,34
Idowu et al. 2011 (3)	0,50	0,14	0,86	2,38
Idowu et al. 2011 (4)	0,50	0,14	0,86	2,38
Idowu et al. 2011 (5)	0,33	-0,03	0,69	2,39
Idowu et al. 2006 (1)	1,00	0,46	1,54	2,17
Idowu et al. 2006 (2)	3,00	2,26	3,74	1,90
Idowu et al. 2006 (3)	2,00	1,38	2,62	2,06
Idowu et al. 2006 (4)	1,00	0,46	1,54	2,17
Idowu et al. 2006 (5)	3,00	2,26	3,74	1,90
Idowu et al. 2006 (6)	0,00	-0,51	0,51	2,21
Venglovská 2014 (1)	1,50	0,86	2,14	2,03
Venglovská 2014 (2)	0,00	-0,57	0,57	2,14
Venglovská 2104 (3)	0,00	-0,57	0,57	2,14
Li et al. 2018 (1)	-0,50	-0,79	-0,21	2,46
Li et al. 2018 (2)	0,00	-0,28	0,28	2,46
Li et al. 2018 (3)	0,00	-0,28	0,28	2,46
Li et al. 2018 (4)	0,50	0,21	0,79	2,46
Stringhini et al. 2015 (1)	0,50	0,21	0,79	2,46
Stringhini et al. 2015 (2)	0,50	0,21	0,79	2,46
Jim et al. 2018 (1)	0,00	-0,35	0,35	2,40
Jim et al. 2018 (2)	0,00	-0,35	0,35	2,40
	0,75	0,55	0,94	

Statistics for each study

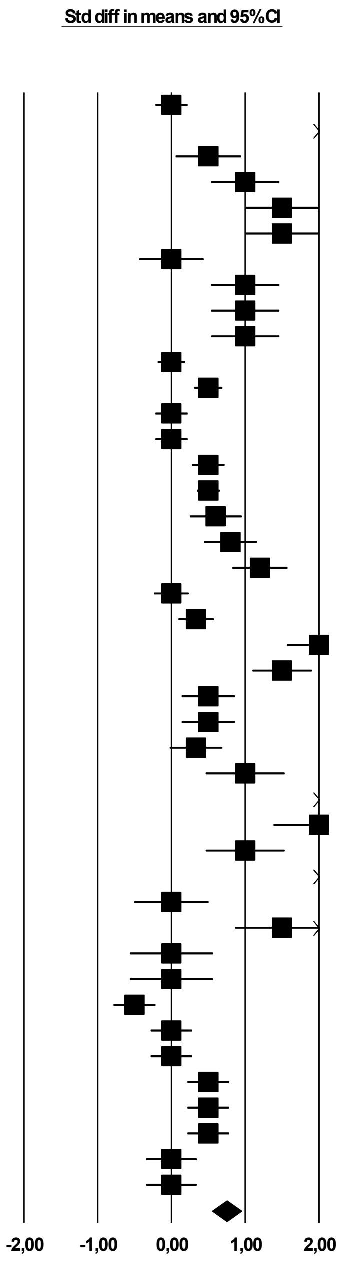

Figure S8: Forest plot of the effect of organic trace mineral supplementation on eggshell thickness (mm) of laying hens. Study name refers to the reference of that study. Raw mean differences (difference in means) represent the effect size estimate. Each square represents the mean effect size for that study. Thus, the squares to the right of the zero mid-line represent higher eggshell thickness, squares to the left of the 0 mid-line indicate lower eggshell thickness and squares at the 0 mid-line indicate no effect on eggshell thickness. The upper and lower limit of the line connected to the square represents the upper and lower 95%

confidence interval (CI) for the effect size. The size of the square reflects the relative weighting of the study to the overall effect size estimate, with larger squares representing greater weight. The diamond at the bottom represents the overall effect size estimate.

Study name	Std diff in means	Lower limit	Upper limit	Relative weight
Stefanello et al. 2014 (1)	0,40	-0,04	0,84	1,84
Stefanello et al. 2014 (2)	0,78	0,32	1,23	1,83
Stefanello et al. 2014 (3)	1,33	0,85	1,82	1,79
Stefanello et al. 2014 (4)	1,62	1,12	2,13	1,77
Stefanello et al. 2014 (5)	0,06	-0,38	0,49	1,84
Stefanello et al. 2014 (6)	0,39	-0,05	0,83	1,84
Stefanello et al. 2014 (7)	0,33	-0,11	0,77	1,84
Stefanello et al. 2014 (8)	0,83	0,38	1,29	1,82
Nolan 2019 (1)	-0,40	-0,80	0,00	1,88
Nolan 2019 (2)	-0,42	-0,83	-0,02	1,88
Nolan 2019 (3)	0,31	-0,09	0,71	1,88
Nolan 2019 (4)	-0,07	-0,47	0,33	1,88
Nolan 2019 (5)	-0,80	-1,22	-0,38	1,87
Nolan 2019 (6)	-0,71	-1,12	-0,30	1,87
Nolan 2019 (7)	-0,76	-1,17	-0,34	1,87
Nolan 2019 (8)	-0,67	-1,08	-0,26	1,87
Ceylan and Scheideler 1999	0,32	-0,14	0,78	1,81
Siske 2000 (1.1)	0,37	0,18	0,56	2,04
Siske 2000 (1.2)	0,39	0,20	0,59	2,04
Siske 2000 (2.1)	-0,19	-0,41	0,03	2,03
Siske 2000 (2.2)	0,05	-0,17	0,27	2,03
Siske 2000 (2.3)	0,08	-0,14	0,30	2,03
Siske 2000 (3)	0,38	0,22	0,53	2,06
Ao et al. 2016 (1)	0,08	-0,22	0,39	1,97
Ao et al. 2016 (2)	0,67	0,36	0,98	1,96
Ao et al. 2016 (3)	0,58	0,27	0,89	1,96
Qiu et al. 2020 (1)	-0,15	-0,39	0,09	2,02
Qiu et al. 2020 (2)	2,74	2,41	3,08	1,94
Yildiz et al. 2011 (1)	2,02	1,14	2,90	1,35
Yildiz et al. 2011 (2)	0,80	0,06	1,55	1,50
Yildiz et al. 2011 (3)	3,36	2,25	4,47	1,11
Yildiz et al. 2011 (4)	5,13	3,64	6,61	0,81
Yildiz et al. 2011 (5)	1,55	0,73	2,37	1,42
Yildiz et al. 2011 (6)	0,57	-0,16	1,30	1,52
Yildiz et al. 2011 (7)	0,56	-0,17	1,29	1,52
Yildiz et al. 2011 (8)	0,70	-0,03	1,44	1,51
Yildiz et al. 2011 (9)	0,42	-0,30	1,15	1,52
Yildiz et al. 2011 (10)	-0,04	-0,76	0,67	1,53
Yildiz et al. 2011 (11)	-0,04	-0,75	0,68	1,53
Yildiz et al. 2011 (12)	-0,01	-0,73	0,70	1,53
Yildiz et al. 2011 (13)	-0,04	-0,76	0,67	1,53
Yildiz et al. 2011 (14)	0,38	-0,34	1,10	1,52
Yildiz et al. 2011 (15)	0,51	-0,21	1,24	1,52
Yildiz et al. 2011 (16)	0,71	-0,03	1,44	1,51
Yildiz et al. 2011 (17)	0,37	-0,35	1,09	1,52
Yildiz et al. 2011 (18)	0,18	-0,53	0,90	1,53
Yildiz et al. 2011 (19)	0,23	-0,49	0,94	1,53
Yildiz et al. 2011 (20)	0,28	-0,44	1,00	1,53
Yildiz et al. 2011 (21)	0,18	-0,54	0,89	1,53
Yildiz et al. 2011 (22)	0,01	-0,70	0,73	1,53
Yildiz et al. 2011 (23)	-0,02	-0,73	0,70	1,53
Yildiz et al. 2011 (24)	0,03	-0,68	0,75	1,53
Yildiz et al. 2011 (25)	0,08	-0,64	0,79	1,53
Li et al. 2018 (1)	-0,40	-0,69	-0,11	1,98
Li et al. 2018 (2)	-0,07	-0,35	0,22	1,98
Li et al. 2018 (3)	-0,40	-0,69	-0,11	1,98
Li et al. 2018 (4)	0,36	0,07	0,64	1,98
Kocher et al. 2020	-0,36	-0,69	-0,02	1,94
	0,34	0,17	0,51	

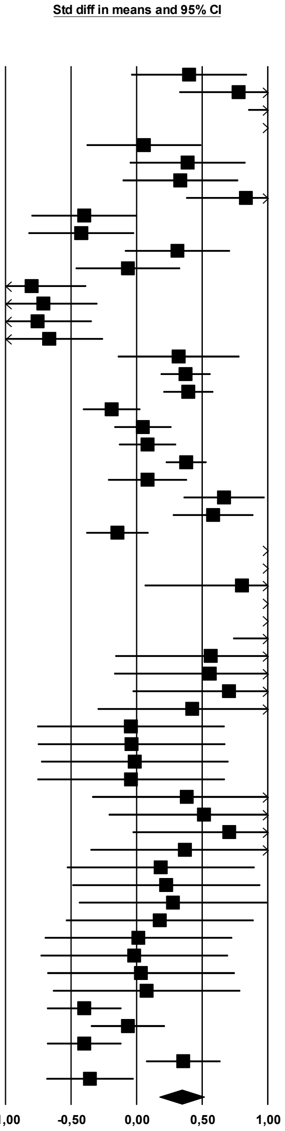

Figure S9: Forest plot of the effect of organic trace mineral supplementation on eggshell strength (kgf) of laying hens. Study name refers to the reference of that study. Raw mean differences (difference in means) represent the effect size estimate. Each square represents the mean effect size for that study. Thus, the squares to the right of the zero mid-line represent higher eggshell strength, squares to the left of the 0 mid-line indicate lower eggshell strength and squares at the 0 mid-line indicate no effect on eggshell strength. The upper and lower limit of the line connected to the square represents the upper and lower 95% confidence interval (CI) for the effect size. The size of the square reflects the relative

weighting of the study to the overall effect size estimate, with larger squares representing greater weight. The diamond at the bottom represents the overall effect size estimate**.**

Study name	Difference in means	Lower limit	Upper limit	Relative weight
Sara et al. 2008	0,69	0,50	0,88	1,74
Siske 2000 (2.1)	0,06	-0,01	0,13	12,55
Siske 2000 (2.2)	0,11	0,04	0,18	12,55
Siske 2000 (2.3)	0,18	0,11	0,25	11,12
Siske 2000 (3)	0,28	0,22	0,34	16,07
Yildiz et al. 2011 (1)	0,12	-0,43	0,67	0,20
Yildiz et al. 2011 (2)	0,06	-0,36	0,48	0,36
Yildiz et al. 2011 (3)	0,02	-0,52	0,56	0,21
Yildiz et al. 2011 (4)	0,15	-0,18	0,48	0,57
Yildiz et al. 2011 (5)	-0,07	-0,40	0,26	0,57
Yildiz et al. 2011 (6)	0,03	-0,30	0,36	0,57
Yildiz et al. 2011 (7)	0,12	-0,28	0,52	0,38
Yildiz et al. 2011 (8)	0,17	-0,19	0,53	0,48
Yildiz et al. 2011 (9)	0,09	-0,19	0,37	0,79
Yildiz et al. 2011 (10)	0,15	-0,49	0,79	0,15
Yildiz et al. 2011 (11)	0,15	-0,34	0,64	0,26
Yildiz et al. 2011 (12)	0,20	-0,24	0,64	0,31
Yildiz et al. 2011 (13)	0,12	-0,24	0,48	0,48
Yildiz et al. 2011 (14)	0,02	-0,67	0,71	0,13
Yildiz et al. 2011 (15)	-0,07	-0,54	0,40	0,28
Yildiz et al. 2011 (16)	0,07	-0,43	0,57	0,25
Yildiz et al. 2011 (17)	-0,01	-0,43	0,41	0,36
Yildiz et al. 2011 (18)	0,10	-0,43	0,63	0,22
Yildiz et al. 2011 (19)	0,01	-0,30	0,32	0,65
Yildiz et al. 2011 (20)	0,10	-0,27	0,47	0,45
Yildiz et al. 2011 (21)	0,07	-0,18	0,32	0,98
Yildiz et al. 2011 (22)	-0,04	-0,65	0,57	0,17
Yildiz et al. 2011 (23)	-0,13	-0,52	0,26	0,41
Yildiz et al. 2011 (24)	-0,04	-0,50	0,42	0,29
Yildiz et al. 2011 (25)	0,01	-0,41	0,43	0,36
Idowu et al. 2011 (1)	1,38	1,16	1,60	1,25
Idowu et al. 2011 (2)	0,35	0,12	0,58	1,14
Idowu et al. 2011 (3)	0,18	-0,11	0,47	0,73
Idowu et al. 2011 (4)	0,61	0,32	0,90	0,72
Idowu et al. 2011 (5)	0,54	0,25	0,83	0,75
Venglovská 2014 (1)	0,58	0,38	0,78	1,49
Venglovská 2014 (2)	0,04	-0,16	0,24	1,49
Venglovská 2104 (3)	0,01	-0,19	0,21	1,49
Mehran Torki 2014	0,52	0,02	1,02	0,25
Stringhini et al. 2015 (1)	0,07	0,00	0,14	13,39
Stringhini et al. 2015 (2)	0,04	-0,03	0,11	13,39

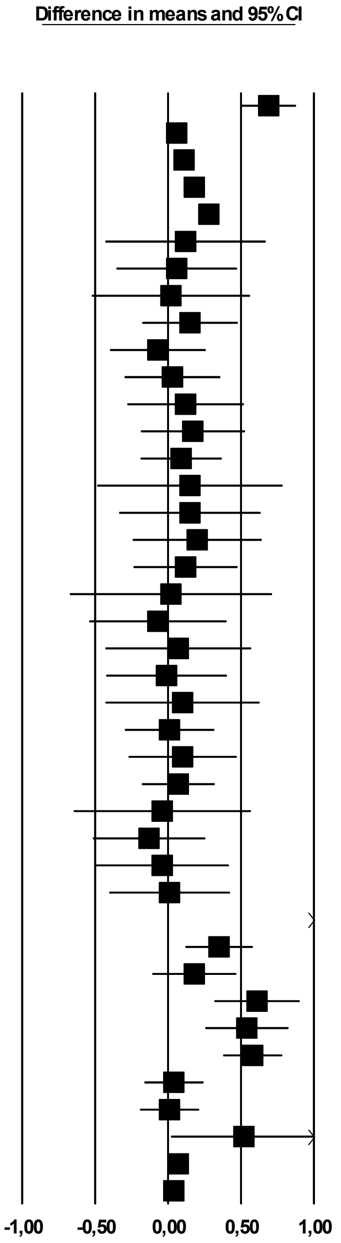

Figure S10: Forest plot of the effect of organic trace mineral supplementation on eggshell weight (g) of laying hens. Study name refers to the reference of that study. Raw mean differences (difference in means) represent the effect size estimate. Each square represents the mean effect size for that study. Thus, the squares to the right of the zero mid-line represent higher eggshell weight, squares to the left of the 0 mid-line indicate lower eggshell weight and squares at the 0 mid-line indicate no effect on eggshell weight. The upper and lower limit of the line connected to the square represents the upper and lower 95%

confidence interval (CI) for the effect size. The size of the square reflects the relative weighting of the study to the overall effect size estimate, with larger squares representing greater weight. The diamond at the bottom represents the overall effect size estimate.

Study name	Difference in means	Lower limit	Upper limit	Relative weight
Murakami and Franco 2004	-0,17	-0,21	-0,13	2,66
Sara et al. 2008	1,00	0,64	1,36	1,58
Ao et al. 2012 (1)	0,17	-0,03	0,37	2,24
Ao et al. 2012 (2)	0,03	-0,17	0,23	2,24
Stefanello et al. 2014 (1)	0,04	-0,14	0,22	2,28
Stefanello et al. 2014 (2)	0,09	-0,09	0,27	2,28
Stefanello et al. 2014 (3)	0,14	-0,04	0,32	2,28
Stefanello et al. 2014 (4)	0,19	0,01	0,37	2,28
Stefanello et al. 2014 (5)	0,00	-0,04	0,04	2,65
Stefanello et al. 2014 (6)	0,06	0,02	0,10	2,65
Stefanello et al. 2014 (7)	0,02	-0,02	0,06	2,65
Stefanello et al. 2014 (8)	0,04	-0,00	0,08	2,65
Nolan 2019 (1)	0,18	0,01	0,35	2,34
Nolan 2019 (2)	0,96	0,79	1,13	2,34
Nolan 2019 (3)	-0,49	-0,66	-0,32	2,34
Nolan 2019 (4)	1,01	0,84	1,18	2,34
Nolan 2019 (5)	1,17	1,00	1,34	2,34
Nolan 2019 (6)	0,26	0,09	0,43	2,34
Nolan 2019 (7)	0,57	0,40	0,74	2,34
Nolan 2019 (8)	-0,91	-1,08	-0,74	2,34
Ceylan and Scheideler 1999	0,15	-0,16	0,46	1,79
Ao et al. 2016 (1)	0,08	-0,06	0,22	2,44
Ao et al. 2016 (2)	0,30	0,16	0,44	2,44
Ao et al. 2016 (3)	0,24	0,10	0,38	2,44
Ao et al. 2018	0,07	0,03	0,11	2,66
Idowu et al. 2011 (1)	0,03	-0,21	0,27	2,08
Idowu et al. 2011 (2)	0,08	-0,15	0,31	2,09
Idowu et al. 2011 (3)	-0,51	-0,81	-0,21	1,82
Idowu et al. 2011 (4)	0,24	-0,06	0,54	1,83
Idowu et al. 2011 (5)	0,28	0,00	0,56	1,92
Idowu et al. 2006 (1)	0,10	0,02	0,18	2,59
Idowu et al. 2006 (2)	0,20	0,11	0,29	2,58
Idowu et al. 2006 (3)	0,03	-0,06	0,12	2,58
Idowu et al. 2006 (4)	0,03	-0,07	0,13	2,55
Idowu et al. 2006 (5)	0,13	0,04	0,22	2,57
Idowu et al. 2006 (6)	-0,07	-0,17	0,03	2,56
Venglovská 2014 (1)	0,88	0,61	1,15	1,93
Venglovská 2014 (2)	0,04	-0,23	0,31	1,93
Venglovská 2104 (3)	0,09	-0,18	0,36	1,93
Stringhini et al. 2015 (1)	0,13	0,04	0,22	2,57
Stringhini et al. 2015 (2)	0,30	0,21	0,39	2,57
Jim et al. 2018 (1)	-0,27	-0,39	-0,15	2,49
Jim et al. 2018 (2)	-0,21	-0,33	-0,09	2,49
	0,15	0,07	0,22	

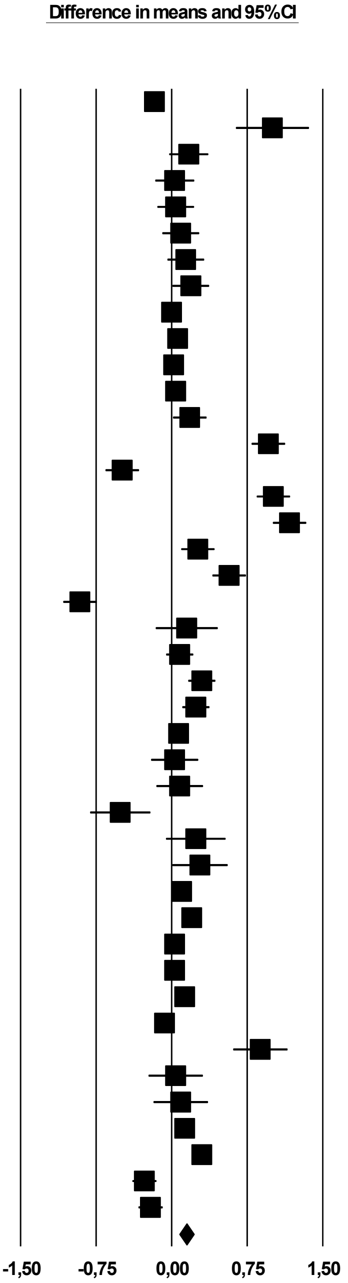

Figure S11: Forest plot of the effect of organic trace mineral supplementation on eggshell percentage (%) of laying hens. Study name refers to the reference of that study. Raw mean differences (difference in means) represent the effect size estimate. Each square represents the mean effect size for that study. Thus, the squares to the right of the zero mid-line represent a higher eggshell percentage, squares to the left of the 0 mid-line indicate a lower eggshell percentage and squares at the 0 mid-line indicate no effect on eggshell percentage.

The upper and lower limit of the line connected to the square represents the upper and lower 95% confidence interval (CI) for the effect size. The size of the square reflects the relative weighting of the study to the overall effect size estimate, with larger squares representing greater weight. The diamond at the bottom represents the overall effect size estimate.

Study name	Std diff in means	Lower limit	Upper limit	Relative weight
Murakami and Franco 2004	0,82	0,59	1,05	2,82
Stefanello et al. 2014 (1)	0,00	-0,44	0,44	2,42
Stefanello et al. 2014 (2)	0,07	-0,37	0,51	2,42
Stefanello et al. 2014 (3)	0,05	-0,38	0,49	2,42
Stefanello et al. 2014 (4)	0,07	-0,37	0,51	2,42
Stefanello et al. 2014 (5)	-0,22	-0,66	0,22	2,42
Stefanello et al. 2014 (6)	0,43	-0,01	0,88	2,41
Stefanello et al. 2014 (7)	0,00	-0,44	0,44	2,42
Stefanello et al. 2014 (8)	0,00	-0,44	0,44	2,42
Nolan 2019 (1)	-0,05	-0,45	0,35	2,50
Nolan 2019 (2)	0,08	-0,32	0,48	2,50
Nolan 2019 (3)	0,09	-0,31	0,49	2,50
Nolan 2019 (4)	0,18	-0,22	0,59	2,50
Nolan 2019 (5)	0,23	-0,17	0,63	2,50
Nolan 2019 (6)	0,05	-0,35	0,45	2,50
Nolan 2019 (7)	0,15	-0,25	0,55	2,50
Nolan 2019 (8)	-0,33	-0,73	0,08	2,50
Carlos and Solano 2005 (trial 1)	0,03	-0,43	0,49	2,37
Siske 2000 (3)	0,36	0,20	0,52	2,92
Qiu et al. 2020 (1)	-0,40	-0,64	-0,16	2,80
Qiu et al. 2020 (2)	0,02	-0,22	0,26	2,81
Idowu et al. 2011 (1)	0,95	0,57	1,33	2,55
Idowu et al. 2011 (2)	0,55	0,18	0,91	2,58
Idowu et al. 2011 (3)	0,56	0,20	0,93	2,58
Idowu et al. 2011 (4)	0,91	0,53	1,28	2,55
Idowu et al. 2011 (5)	1,32	0,93	1,72	2,51
Idowu et al. 2006 (1)	1,80	1,20	2,40	2,07
Idowu et al. 2006 (2)	2,07	1,45	2,70	2,01
Idowu et al. 2006 (3)	-0,25	-0,76	0,25	2,27
Idowu et al. 2006 (4)	-0,45	-0,97	0,06	2,26
Idowu et al. 2006 (5)	0,03	-0,48	0,53	2,28
Idowu et al. 2006 (6)	-0,73	-1,25	-0,21	2,24
Mehran Torki 2014	0,30	-0,21	0,81	2,27
Li et al. 2018 (1)	-0,16	-0,44	0,12	2,73
Li et al. 2018 (2)	0,04	-0,24	0,32	2,73
Li et al. 2018 (3)	0,02	-0,26	0,30	2,73
Li et al. 2018 (4)	0,02	-0,27	0,30	2,73
Kocher et al. 2020	1,41	1,04	1,78	2,57
Jim et al. 2018 (1)	-0,22	-0,57	0,12	2,61
Jim et al. 2018 (2)	-0,18	-0,52	0,17	2,61
	0,23	0,08	0,39	

Statistics for each study

Std diff in means and 95% CI

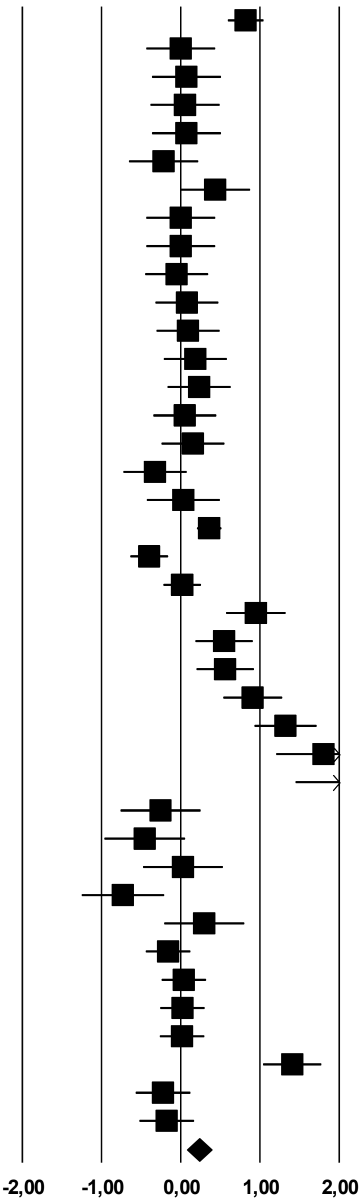

Figure S12: Forest plot of the effect of organic trace mineral supplementation on Haugh unit of laying hens. Study name refers to the reference of that study. Raw mean differences (difference in means) represent the effect size estimate. Each square represents the mean effect size for that study. Thus, the squares to the right of the zero mid-line represent a higher Haugh unit, squares to the left of the 0 mid-line indicate a lower Haugh unit and squares at the 0 mid-line indicate no effect on Haugh unit. The upper and lower limit of the line

connected to the square represents the upper and lower 95% confidence interval (CI) for the effect size. The size of the square reflects the relative weighting of the study to the overall effect size estimate, with larger squares representing greater weight. The diamond at the bottom represents the overall effect size estimate.
