# Supplementary material for: The Effect of Organic Trace Mineral Supplementation in the Form of Proteinates on Performance and Sustainability Parameters in Laying Hens: A Meta-Analysis
Source: Animals (Basel). 2023 Oct 7;13(19):3132. doi: 10.3390/ani13193132 (PMC10571535; doi:10.3390/ani13193132)
Supplement: Supplementary file 1 [file animals-13-03132-s001.zip › OTM supplementary tables S1 to S3.pdf]

Table S1. Ingredient composition of formulated low- and high-soybean meal (SBM) diets in different laying phases

Ingredient (%)	Low-SBM diet				High-SBM diet			
	Pre-lay	Early-lay	Mid-lay	Late-lay	Pre-lay	Early-lay	Mid-lay	Late-lay
Wheat	62.53	54.75	59.49	62.36	62.53	53.66	58.07	62.36
Wheat feed	10.00	10.00	10.00	10.00	10.00	6.47	10.00	10.00
Soybean meal[1]	15.21	15.34	6.52	3.60	15.21	24.35	9.35	3.60
Sunflower meal[2]	5.00	5.80	10.00	10.00	5.00	-	8.25	10.00
Soybean oil	1.13	3.59	3.55	3.32	1.13	3.66	3.60	3.32
Limestone	4.13	8.81	8.93	9.27	4.13	9.75	9.17	9.27
Monocalcium phosphate	1.00	0.70	0.47	0.37	1.00	0.96	0.53	0.37
Sodium chloride	0.26	0.26	0.21	0.21	0.26	0.29	0.24	0.21
Sodium bicarbonate	0.15	0.15	0.15	0.15	0.15	0.17	0.15	0.15
Lysine-HCl	0.02	-	0.09	0.14	0.02	-	0.06	0.14
DL-methionine	0.05	0.09	0.06	0.05	0.05	0.18	0.07	0.05
Synergen	0.02	0.01	0.01	0.01	0.02	0.02	0.02	0.01
Vitamin-mineral premix	0.50	0.50	0.50	0.50	0.50	0.50	0.50	0.50
Total	100	100	100	100	100	100	100	100

[1]Soybean meal contains 46.5% crude protein.
[2]Sunflower meal contains 36% crude protein.

Table S2. Summary of daily feed intake, crude protein content and estimated global warming potential (GWP) of formulated low- and high-soybean meal (SBM) diets

	Low-SBM diet			High-SBM diet		
Diet	Feed intake (g/day/hen)	Crude protein (%)	GWP (g CO_2-eq/kg)	Feed intake (g/day/hen)	Crude protein (%)	GWP (g CO_2-eq/kg)
Pre-lay	100	17.5	1027	100	17.5	1027
Early-lay	110	17.0	1060	100	18.5	1485
Mid-lay	120	15.0	649	115	15.5	780
Late-lay	120	14.0	508	120	14.0	508

Table S3. Data describing production characteristics and laying performance of baseline and organic trace mineral (OTM, Bioplex Cu, Fe, Mn, Zn) scenarios

**Item**	**Baseline**	**OTM**
Laying cycle (week)	62	62
Hen-day production (%)	85	87
Eggs produced per bird (n)	369	378
Egg weight (g)	65	65.5
Total egg mass per hen (kg)	23.99	24.73
Mortality (%)	3.50	3.50
Egg loss (%)	1.5	0.9
Total feed per hen (kg)	50.35	49.13
FCR (g feed/kg egg)	2100	2049
